# Unlocking the Pharmacological Potential of Myricetin Against Various Pathogenesis

**DOI:** 10.3390/ijms26094188

**Published:** 2025-04-28

**Authors:** Saleh A. Almatroodi, Arshad Husain Rahmani

**Affiliations:** Department of Medical Laboratories, College of Applied Medical Sciences, Qassim University, Buraydah 51452, Saudi Arabia

**Keywords:** myricetin, oxidative stress, inflammation, pathogenesis, nanoformulation

## Abstract

Myricetin is a natural flavonoid with powerful antioxidant and anti-inflammatory potential commonly found in vegetables, fruits, nuts, and tea. The vital role of this flavonoid in the prevention and treatment of various diseases is evidenced by its ability to reduce inflammation and oxidative stress, maintain tissue architecture, and modulate cell signaling pathways. Thus, this review summarizes recent evidence on myricetin, focusing precisely on its mechanisms of action in various pathogenesis, including obesity, diabetes mellitus, arthritis, osteoporosis, liver, neuro, cardio, and reproductive system-associated pathogenesis. Moreover, it has been revealed that myricetin exhibits anti-microbial properties due to obstructive virulence factors, preventing biofilm formation and disrupting membrane integrity. Additionally, synergistic potential with other drugs and the role of myricetin-based nanoformulations in different diseases are properly discussed. This review seeks to increase the understanding of myricetin’s pharmacological potential in various diseases, principally highlighting its effective mechanisms of action. Further wide-ranging research, as well as more randomized and controlled clinical trial studies, should be executed to reconnoiter this compound’s therapeutic value, safety, and usefulness against various human pathogenesis.

## 1. Introduction

Medicinal plants are commonly used in traditional cultures globally and are gaining acceptance in modern society as natural alternatives or complements to synthetic chemicals [1]. Moreover, medicinal plants are indispensable in curing most pathogeneses, as numerous human illnesses are treated with medications derived from plant components [2]. An estimated 80% of the worldwide population uses traditional medicine to treat as well as manage various diseases [3].

Medicinal plants are abundant in flavonoids, polyphenols, vitamins, and proteins, all significantly contributing to disease management. Additionally, natural products have been vital in the discovery of drugs, especially in the fields of cancer and infectious diseases [4,5]. In addition, natural products and their bioactive ingredients play a substantial role in disease management through different mechanisms [6,7,8,9,10]. Moreover, natural compounds and their bioactive constituents are abundant sources of polyphenols, phenolic acids, carotenoids, vitamins, minerals, and other nutrients, and all these constituents hold disease-management properties. Including these health-boosting substances in diets can provide better health and wellness. Flavonoids represent an important class of secondary metabolites that can be found across a wide range of sources, such as fruits, vegetables, grains, herbs, stems, seeds, nuts, and flowers [11]. More than 10,000 flavonoid compounds have been extracted and recognized [12]. Research has shown that foods high in flavonoids prevent disease development and progression. These foods/flavonoids possess numerous benefits, including anti-analgesic, anti-inflammatory, anti-cancer, anti-microbial, anti-proliferative, and neuroprotective properties [12,13,14].

Myricetin is a natural flavonoid derived from several plant sources, and its role in pathogenesis has been confirmed through different mechanisms of action, mainly through antioxidant and anti-inflammatory potential [15]. The pathogenesis prevention potential is described as myricetin, which was shown to restore the activity of the antioxidant enzymes. Additionally, the compound reduced lipid peroxidation caused by cisplatin, as well as the increase in xanthine oxidase activity and the activity of phase-II detoxifying enzymes. Myricetin also mitigated the damaging effects of cisplatin by regulating the levels of molecular inflammation markers (NF-κB, Nrf-2, TNF-α and IL-6), restoring Nrf-2 levels, and preventing the disintegration of goblet cells [16]. The study was designed to examine the beneficial role of myricetin in a diet-induced nonalcoholic steatohepatitis (NASH) model. C57BL/6J mice were administered either a standard chow diet or a choline-deficient, L-amino acid-defined, high-fat diet (CDAHFD) for a duration of 8 weeks. During this period, they received daily treatment with either myricetin (at a dose of 100 mg/kg) or vehicle by daily gavage. It was reported that myricetin ameliorated hepatic inflammation, steatosis, and inhibited hepatic macrophage infiltration in CDAHFD-fed mice. Also, myricetin-treated to CDAHFD-fed mice prevented liver fibrosis and hepatic stellate cell (HSC) activation as compared to vehicle-treated mice. Furthermore, myricetin inhibited M1 macrophage polarization as well as its relative markers in livers of NASH mice while causing the M2 polarization induction. Likewise, in in vitro findings, myricetin prevented the lipopolysaccharide (LPS)-induced mRNA expression of M1 macrophages marker genes as well as induced IL-4-induced M2 macrophage marker genes in RAW264.7 macrophages. Moreover, myricetin inhibited the expression of TLR2/4-MyD88 and triggering receptor expressed on myeloid cells-1 (TREM-1) signaling molecules in livers from NASH mice as well as in RAW264.7 macrophages stimulated by LPS [17].

Myricetin’s role in cancer is described as myricetin activating ferroptosis in gastric cancer cells by enhancing malondialdehyde production and Fe^2+^ accumulation while suppressing glutathione levels. Moreover, in vivo experiments confirmed that myricetin treatment meaningfully inhibited the growth of subcutaneous tumors in BALB/c nude mice [18]. Its role as a hepatoprotective was noticed as myricetin prevented hepatotoxicity via modulating the ethanol metabolizing enzymes, production of free radicals, and inflammatory markers in vivo. Myricetin maintains oxidant–antioxidant status, lipid membrane integrity, and histoarchitecture. After ethanol administration, there was an increase in the aspartate aminotransferase (AST), aspartate transaminase (AST), malondialdehyde (MDA); myricetin administration reduced the level of all these markers. Additionally, treatment of myricetin decreased ethanol-induced inflammatory markers [19]. This review described the role of myricetin in pathogenesis and how it influences different biological activities. Additionally, it discusses the synergistic effects when combined with other compounds, as well as the potential impact of nanoformulations in various pathogenesis.

## 2. Methodology

Search engines such as PubMed, Scopus, Google Scholar, and Web of Science were used to collect data concerning myricetin and its role in pathogenesis management. The keywords used to collect data included sources, intake of myricetin, oxidative stress, inflammation, cardioprotective effects, neuroprotective potential, and role in the respiratory and reproductive systems. Its role is in anti-obesity, wound healing, anti-cancer, bone disease, and inhibition of microorganisms. Nanoformulation based on myricetin and synergistic effect with other compounds was searched. The literature that was comprised of experimental findings, randomized controlled trials, and epidemiological studies associated with myricetin was included in this study, whereas case reports, editorials, and thesis were excluded.

## 3. Structure, Sources, Daily Intake, and Pharmacokinetics of Myricetin

**Figure 1:**
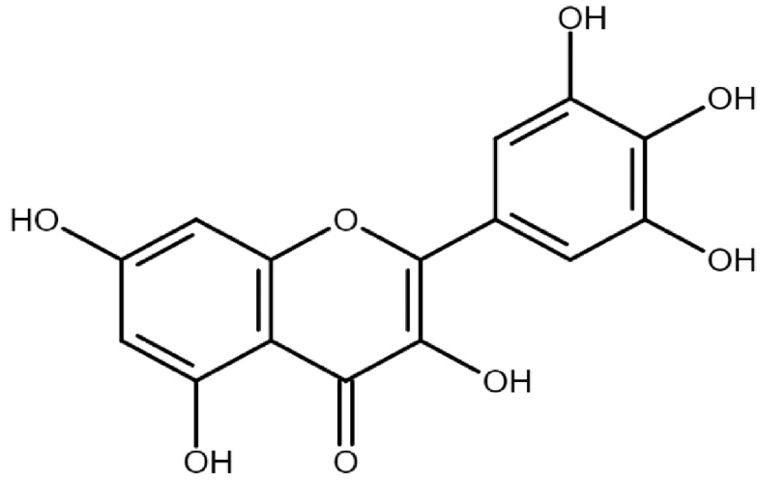
Chemical structure of myricetin (Chemical structure was made using the Chemical Sketch Tool: https://www.rcsb.org/chemical-sketch).

Myricetin (3,5,7,3′,4′,5′-hexahydroxyflavone), with the chemical formula C_15_H_10_O_8_, has a relative molecular mass of 318.24. It is a member of the class of flavonoids/plant-derived flavonoids known as flavonols. This flavonoid is usually found in nature within berries, fruits and vegetables, typically as glycosides instead of free aglycones [20]. Additionally, flavonoids are predominantly found in their glycoside form in a variety of sources such as nuts, vegetables, herbs, fruits, plants, and beverages like tea, fruit juices, wine, and medicinal plants [21,22,23,24,25,26,27]. The amount present in various fruits and vegetables varies within different ranges. The quantities of myricetin in black fruits ranges between 14 and 142 mg/kg [24], and in honey, it ranges from 29.2 to 289 μg/100 g honey [28]. A quantity of 57.2 mg/g of myricetin per gram was extracted from the fruits of *Lycium barbarum* L. [29], while the skin of *Carménère grapes* contains 2.4 mg of myricetin per kg [30]. The concentration of myricetin in plants like *Rosa canina* L. (rose hip), *Urtica dioica* L. (nettle), and *Portulaca oleracea* L. (purslane) ranges from 3 to 58 mg/kg [25]. The estimated daily intake of myricetin is approximately 1.1 mg for males and around 0.98 mg for females [31]. The average daily consumption of myricetin is 2.2 ± 2.5 mg [32]. The average intake of myricetin for adults aged 18 to 64 years in the European Union is 2 mg per day, ranging between 1 and 4 mg per day [33]. The chemical structure of myricetin is shown in Figure 1.

Myricetin’s influence on the pharmacokinetics of losartan and its active metabolite, EXP-3174, was studied in rats. The pharmacokinetic parameters for both losartan and EXP-3174 were assessed following the oral administration of losartan (9 mg/kg) to the rats, either with or without the presence of myricetin (at doses of 0.4, 2, and 8 mg/kg). Moreover, myricetin notably increased the cellular uptake of rhodamine 123 in MCF-7/ADR cells that overexpress P-glycoprotein, and this effect was dependent on the concentration. The pharmacokinetic parameters of losartan were significantly modified by myricetin in comparison to the control group. The addition of myricetin (at doses of 2 or 8 mg/kg) resulted in a 31.4–61.1% increase in the area under the plasma concentration-time curve for losartan, as well as a 31.8–50.2% rise in the peak plasma concentration of losartan. Accordingly, the absolute bioavailability of losartan in the presence of myricetin increased significantly compared with the control. As a result, the absolute bioavailability of losartan was significantly enhanced in the presence of myricetin compared to the control group. Moreover, the simultaneous administration of myricetin (8 mg/kg) led to a substantial 20% decrease in the ratio of the area under the plasma concentration-time curve for the metabolite to that of the parent drug. This advocates that myricetin might inhibit the cytochrome P450 (CYP)-mediated metabolism of losartan to its active metabolite (EXP-3174) [34]. A particular and precise ultra-performance liquid chromatography-tandem mass spectrometry (UPLC-MS/MS) technique was established and verified for the detection and measurement of myricetin in rat plasma following both oral and intravenous administration. Followed by β-glucuronidase as well as sulfatase hydrolysis and liquid-liquid extraction with ethyl acetate, the analytes were separated on an Acquity UPLC BEH C18 column and studied in the selected ion recording with a negative electrospray ionization mode. The method was successfully employed to conduct a pharmacokinetic study of myricetin following both intravenous and oral administration in rats. The absolute bioavailability was found to be 9.62% for the 50 mg/kg dose and 9.74% for the 100 mg/kg dose, indicating that myricetin has limited absorption when administered orally [35].

## 4. Effects of Myricetin on Human Health

Scientific studies have established that myricetin offers various health benefits by modulating multiple biological activities. These benefits are mainly attributed to its anti-inflammatory and antioxidant properties, which play a significant role in the body’s response to stress as well as disease. The biological activities of myricetin are outlined below.

### 4.1. Antioxidant Potential

Oxidative stress (OS) is an imbalance between pro-oxidants and antioxidants, leading to dysregulation of redox processes and damage to macromolecules. Oxidative stress has harmful effects as free radicals interact with essential cellular components, for example lipids, proteins, and DNA [36]. Lipid peroxidation disrupts cell membranes, and oxidation of proteins leads to loss of function and structural integrity. Additionally, damage to DNA can lead to mutations and genomic instability [37].

Natural compounds play a substantial role in preventing pathogenesis through its antioxidant potential as well as reducing the oxidative stress [38,39,40]. Moreover, myricetin shows role in disease management through inhibition of oxidative stress related parameters. Supplementation of rat hepatocyte cultures with the myricetin directed to the formation of phenoxyl radical intermediates, as noticed in intact cells. These radicals corresponded to one-electron oxidation products of myricetin. The level of phenoxyl radicals was meaningfully decreased when myricetin-treated hepatocyte cultures were also supplemented with ferric iron nitrilotriacetate (Fe-NTA). This proposed that iron might accelerate the oxidation flux of myricetin. Furthermore, myricetin was found to be capable of preventing lipid peroxidation induced by iron in hepatocyte culture. Free malondialdehyde levels as well as the number of radicals derived from oxidized lipids were significantly decreased when myricetin was added to iron-treated cultures [41]. The antioxidant potential of myricetin on 5-fluorouracil (5-FU)-induced hepatotoxicity was examined. It was reported that the level of MDA was increased in those rats receiving the single intraperitoneal (IP)-dose of 5-FU as compared to the control group. Whereas those rats receiving the lower dose of myricetin alone caused an important reduction in MDA as compared to control group, that same difference does not appear amongst the higher dose of myricetin group. However, both groups of rats receiving both myricetin and 5-FU showed meaningfully reduced levels of MDA compared to 5-FU-only group. On the other hand, there was an important decrease in the hepatic tissue homogenate level of the superoxide dismutase (SOD) enzyme in the 5-FU-only group, whereas those rats receiving myricetin caused an important increase in SOD. The myricetin + 5-FU groups showed SOD levels that were meaningfully higher than those in the 5-FU-only group. Moreover, in the myricetin + 5-FU groups, a substantial increase in the levels of glutathione (GSH), as well as catalase (CAT), was noticed as compared to those in the 5-FU-only group [42].

The study examined the effects of toxic iron chelate, Fe-NTA, on oxidative DNA damage. This investigation was conducted both in the presence and absence of the myricetin at a concentration of 25–50–100 microM. The simultaneously increasing concentrations of myricetin, in addition to iron, effectively prevented lipid peroxidation [43]. The role of myricetin in colon toxicity was examined, and the effects of myricetin on cisplatin-caused colon toxicity were measured in terms of phase-II detoxification and enzyme antioxidant status. The results revealed that myricetin was found to restore the level of all the antioxidant enzymes, ameliorate lipid peroxidation, increase phase-II detoxifying enzyme activity, and increase xanthine oxidase activity [16]. Another study based on liver failure in mice reported that myeloperoxidase (MPO) and MDA levels were reduced, and SOD and CAT activities were improved with myricetin at doses of 50 and 100 mg/kg pretreatment. These findings suggest that myricetin protects against liver failure by regulating oxidative stress via Nrf2 signaling and that it might be a probable approach to avoid liver damage [44]. Abolfazl Barzegar 2016 demonstrated that intracellular reactive oxygen species (ROS) are highly toxic, and applying low concentrations of myricetin inhibited the cellular production of ROS. Because of the excellent correlation between cellular reactive oxygen species and their cell toxic effects, the higher antioxidant effectiveness of myricetin caused an efficient inhibited intracellular ROS and defended against cell death [45]. Treatment with aluminum phosphide increased cytotoxicity, cellular ROS formation, ATP depletion, and MDA level, and decreased the activities of antioxidant enzymes in cardiomyocytes. It was noticed that myricetin at a dose of 80 µM demonstrated ameliorated aluminum phosphide-caused cytotoxicity in isolated cardiomyocytes, meaningfully decreasing the aluminum phosphide-stimulated MDA production, intracellular ROS, and reduction of GSH, and increased the activities of SOD, catalase, and glutathione peroxidase (GSH-Px) [46]. The protective role of myricetin against tert-butylhydroperoxide (t-BHP) induced oxidative stress in human erythrocytes was examined. Incubating erythrocytes with t-BHP caused development of oxidative stress, as shown by enhancement in erythrocyte MDA and protein carbonyl content, and a reduction in intracellular-reduced GSH membrane sulphydryl (-SH) groups. Incubation of erythrocytes by myricetin, at the same time as t-BHP, protected the erythrocytes from oxidative stress [47]. A study was performed to reconnoiter the regressive effect of myricetin on pre-existing hepatic steatosis caused by a high-fat diet (HFD). Myricetin treatment meaningfully alleviated HFD-induced steatosis, reduced hepatic lipid accumulation, increased antioxidative enzyme activities and thiobarbituric acid reactive substance (TBARS) levels. Microarray analysis of hepatic gene expression profiles presented that myricetin suggestively altered the expression profiles of 177 genes which were participated in 12 biological pathways, with the peroxisome proliferator activated receptor (PPAR) signaling pathway as well as peroxisome [48]. The potential effect of myricetin on obesity was investigated. It was demonstrated that obesity-associated oxidative stress (total antioxidant capacity, glutathione peroxidase activity), malondialdehyde, and inflammation were ameliorated in mice treated with myricetin [49]. Another experiment was performed to inspect the hepatoprotective potential of myricetin against high-fat diet-induced hepatic steatosis mice, and 0.12% w/w myricetin in the diet was given to animals. Myricetin reduced HFD-induced steatosis, lipid accumulation, and TBARS levels and enhanced the glutathione peroxidase, SOD, and catalase antioxidant enzyme activities in the liver [50].

A vital study result reported that myricetin suggestively bettered diabetes-induced impairment in sensation, nerve blood flow, and nerve conduction velocities; furthermore, myricetin significantly decreased the generation of advanced glycation end-products (AGEs), elevated Na+, K+-ATPase activity and ROS, and antioxidant activities in nerves in diabetic animals. In addition, studies revealed that myricetin meaningfully elevated the hydrogen sulfide (H_2_S) levels and elevated the expression level of nuclear factor-E2-related factor-2 (Nrf2) as well as heme oxygenase-1 (HO-1) in diabetic rats [51].

### 4.2. Anti-Inflammatory Effects

Flavonoids and natural compounds exhibited anti-inflammatory effects [52,53,54] by reducing the production of pro-inflammatory agents and downregulating cyclooxygenase-2 (COX-2) expression. Moreover, the flavanols have confirmed the ability to inhibit lipoxygenase [55]. This action reduces the production of key inflammatory substances, including prostaglandins, leukotrienes, and nitric oxide [56]. Flavonoids also diminish the release of metabolites from arachidonic acid and chemokines, which helps to reduce leukocyte infiltration and edema [57]. Furthermore, flavonoids can chelate iron and inhibit the activation of the complement system, contributing to a decrease in inflammation [58].

Myricetin revealed its anti-inflammatory potential by reducing the production of pro-inflammatory agents and downregulating COX-2, inducible nitric oxide synthase (iNOS) expression, and other mechanisms. Myricetin reduced the production of pro-inflammatory mediators in LPS-stimulated RAW264.7 macrophages in a dose-dependent manner. The administration of myricetin also led to a decrease in the levels of iNOS, NO, IL-6, TNF-α, and IL-12 in mice. Furthermore, myricetin inhibited NF-κB activation by preventing the degradation of IκBα, nuclear translocation of the p65 subunit of NF-κB, and NF-κB DNA binding activity in LPS-stimulated RAW264.7 macrophages. Furthermore, myricetin reduced the phosphorylation of STAT1 as well as the production of IFN-β in LPS-stimulated RAW264.7 macrophages [59]. The effects of myricetin on the expression of cyclooxygenase 2 COX-2 in H9c2 cells treated with peptidoglycan (PGN) from *Streptococcus sanguinis*, a bacterial constituent of dental plaque linked with infective endocarditis, was investigated. Myricetin exposure caused in dose-dependent decrease of PGN-induced expression of COX-2 reduced phosphorylation of p38, extracellular signal regulated kinase 1/2, as well as c-Jun N-terminal kinase, and reduced IκB-α degradation, consistent with COX decreased -2 activity. The results advocate that myricetin is valuable in moderating the inflammatory response in infective endocarditis [60]. The potential of myricetin to modulate LPS-stimulated activation of mouse bone marrow-derived dendritic cells (DCs) was evaluated. It was reported that myricetin significantly reduced the secretion of tumor necrosis factor-α, interleukin-6, and interleukin-12p70 by LPS-stimulated DCs. Myricetin showed a role in the inhibition of expression of LPS-induced major histocompatibility class II, CD40, as well as CD86 on DCs, and the migratory and endocytic capacity of LPS-stimulated DCs was blocked by myricetin. Furthermore, by myricetin, LPS-stimulated DC-elicited allogeneic T-cell proliferation was reduced. Moreover, this outcome established that myricetin reduces the responses of LPS-stimulated activation of DCs through suppression of IκB kinase/nuclear factor-κB as well as mitogen-activated protein kinase-dependent pathways [61]. A study was made to explore whether myricetin was effective in improving sepsis-induced myocardial dysfunction in cardiomyocyte injury. Myricetin considerably produces inflammatory cytokines both in serum and cardiac tissue and inhibits degradation of IκBα, nuclear translocation of p65, and cellular apoptosis. This compound prevented the reduction of oxidoreductase activity and overexpression of iNOS. Furthremore, myricetin treatment could decrease inflammatory cytokines production of peritoneal macrophages stimulated with LPS in vitro [62]. This compound inhibits the upregulation of COX-2 by blocking IκB/NFκB, Akt, and mammalian target of rapamycin (mTOR) signaling. It also decreases the production of cytokines and chemokines in keratinocytes, ultimately mitigating skin inflammation caused by TNF-α and ultraviolet light [63,64]. An interesting study result reported that myricetin dose-dependently inhibited the production of pro-inflammatory mediators in LPS-stimulated RAW264.7 macrophages. Myricetin treatment decreased the production of tumor necrosis factor alpha (TNF-α), Interleukin- 6,12 (IL-6,12), nitric oxide (NO), and iNOS, in mice [59]. Pretreatment with myricetin and its effects on decreased the up-regulation of matrix metalloproteinase 1 (MMP-1) and IL-6, as well as inhibiting the phosphorylation of p38 and JNK in human synovial sarcoma cells in vitro [65]. The effects of apigenin and myricetin against cisplatin-induced nephrotoxicity were examined. Treatment of mice with cisplatin demonstrated a substantial increase in serum levels of IL-6 and TNFα. Then again, treatment with myricetin apigenin, or their combination, improved the above-mentioned increase in serum levels of TNFα [66]. An experiment was performed to explore the role of myricetin in colon toxicity and it was reported that myricetin reduced deteriorative effects by regulating the level of molecular markers of inflammation and controlling goblet cell disintegration [16]. Myricetin significantly reduced lung inflammation as marked by the reduced concentration of protein in the bronchoalveolar lavage fluid (BALF), wet-to-dry weight ratio of lungs, activity of MPO, cytokine production, and inflammatory cell migration. Moreover, the reduction was also noticed in toll-like receptor 4 (TLR4), nuclear factor-kappa B (NF-κB), and MyD88 expression [67].

The extracts as well as active compounds of Chinese bayberry were used to check the chemical antioxidant potential and anti-inflammatory activities in Propionibacterium acnes (*P. acnes*)-stimulated human SZ95 sebocytes. Consequently, the flavonols, myricetin and myricitrin, were found to be rich in the unhydrolyzed and hydrolyzed extracts of Chinese bayberry fruits, correspondingly. The anthocyanin cyanidin-3-glucoside was also mainly found in the unhydrolyzed extracts. Quantification of human inflammatory cytokines designated that cell-free extracts of *P. acnes* stimulated IL-8 and IL-6 production, which was prevented by myricetin. Myricetin also showed inhibitory effects in *P. acnes*-stimulated gene expression of TLR2 as well as protein phosphorylation of p70 S6 kinase [68]. Cells treated with myricetin prevented anti-Fas IgM-induced apoptosis and blocked the synergetic effect of anti-Fas IgM with TNF-alpha or IL-1beta on cell death [69]. A pioneer study reported that myricetin effectively reduced the expression of several indicators of the neuroinflammatory response in LPS-induced activated microglia [70]. Myricetin effectively enhanced the inflammatory response by blocking the AKT/IKK/NF-κB signaling pathway and restoring the integrity of the blood–milk barrier in mice with LPS-induced mastitis [71].

### 4.3. Hepatoprotective Effects

**Figure 2:**
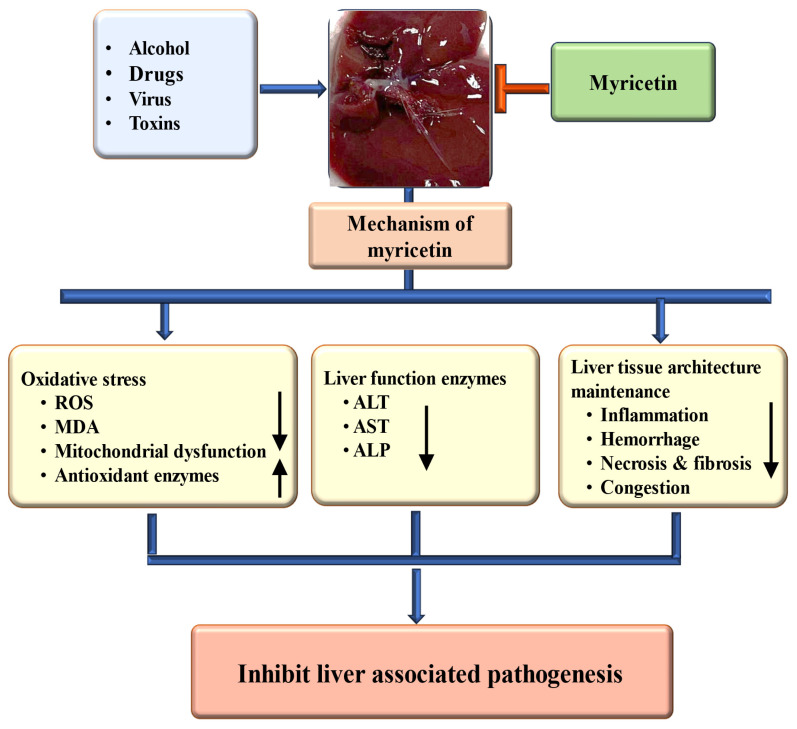
Hepatoprotective potential of myricetin through different mechanisms (the liver image used in the figure is from our lab). The downward-pointing arrow shows downregulation, whereas the upward arrow represents upregulation.

Liver diseases contribute significantly to global rates of illness and death [72]. Chronic liver diseases have become a substantial cause of death and morbidity over the last decade, leading to approximately two million deaths each year worldwide [73]. The primary contributors to chronic liver disease include non-alcoholic fatty liver disease, hepatitis B and C, cirrhosis, and hepatocellular carcinoma. These conditions, along with their complications, play a major role in liver-related mortality [74]. Various types of drugs, including synthetic drugs, are used to treat this pathogenesis. Unfortunately, such treatment leads to negative side effects. Various natural compounds and their bioactive constituents have been confirmed to show a hepatoprotective role by reducing oxidative stress and inflammation and maintaining liver tissue morphology [75,76,77]. The role of myricetin in the inhibition of liver pathogenesis through modulation of different mechanisms is presented in Figure 2.

Moreover, the role of myricetin as a hepatoprotective is summarized in Table 1 as per the previous studies. To explore the role of myricetin in vivo, carbon tetrachloride (CCl4) was induced to develop liver fibrosis. Hematoxylin and eosin (H&E), and Sirius-red staining reported a noticeable increase in the amount of liver fibrosis. Myricetin treatment reduced the amount of liver fibrosis and ameliorated the increase in serum ALT and AST activity caused by CCl4. The anti-fibrotic potential of myricetin in CCl4-treated mice was reported by measuring the effects of myricetin on α-smooth muscle actin (α-SMA) and collagen type I (Col1) protein expression. The Col1 and α -SMA expression were meaningfully reduced in fibrotic mice treated with myricetin [78]. Study findings described that myricetin improved high-fat diet (HFD)-induced hepatic steatosis and TH levels, increased hepatic type 1 deiodinase activities, and elevated energy expenditure concerning the HFD mice. At the same time, myricetin inhibite the upregulation of miR-205 and miR-146b caused by a HFD [79]. Another study finding exhibited that myricetin efficiently protects from LPS/D-GalN-induced fulminant hepatitis by lowering AST and ALT levels and improving oxidative stress, histopathological changes, hepatic apoptosis, and inflammation; furthermore, myricetin efficiently mediates multiple signaling pathways. Myricetin relieved hepatotoxicity excited by H_2_O_2_ and was inhibited by Nrf2-null and AMPK inhibitors [80]. Myricetin decreased the fatality rate of animals, pathological liver changes, improved liver function enzymes (ALT, AST, and ALP), decreased apoptotic, inflammatory factors, and oxidative, and enhanced some antioxidants. In addition, myricetin improved the activity of sirtuin 1 and hepatic level and reversed inappropriate alterations of autophagic parameters [81]. Myricetin inhibited hepatotoxicity by modulating the production of free radicals and inflammatory markers in vivo. Myricetin maintained oxidant–antioxidant status, lipid membrane integrity, and liver tissue architecture. Administration of ethanol increases the hepatotoxicity biomarkers, and myricetin administration reduces the level of these markers [19]. The role of myricetin supplementation on HFD-induced nonalcoholic fatty liver disease (NAFLD) in rats was examined. The 12-week supplementation with myricetin and fecal microbiota transplantation indicates that myricetin significantly decelerates the progression of NAFLD. Myricetin reduces hepatic lipid synthesis and inflammation by modulating the gut microbiota related to fecal butyric acid [82]. Another finding reported that myricetin and apigenin pretreatments increased liver GSH levels and CAT and SOD activities, albumin and total protein levels, and decreased serum liver function enzymes in LPS-treated mice.

Myricetin and apigenin administration also maintained the hepatic architecture disrupted during LPS administration [83]. Xia Wang et al., 2023 reported as myricetin pretreatment decreased serum ALT and AST and improved the pathological changes of liver tissues induced by lipopolysaccharide (LPS)/D-galactosamine (D-Gal). Furthermore, MPO and MDA levels were reduced, SOD and CAT activities were increased with myricetin (50 and 100 mg/kg) pretreatment [44]. The beneficial effects of myricetin against non-alcoholic fatty liver disease were noticed by the regulation of hepatic lipid metabolism transcription factors, pro-inflammatory cytokines, and the antioxidant system [84]. Myricetin improved hepatic inflammation and steatosis and inhibited hepatic macrophage infiltration in CDAHFD-fed mice [17]. Sheikh Bilal Ahmad et al., 2022 reported that myricetin prevented hepatotoxicity by modifying the production of free radicals and inflammatory markers in vivo. Myricetin maintained oxidant–antioxidant status, lipid membrane integrity, and liver tissue architecture [19].

### 4.4. Anti-Diabetic Potential

**Figure 3:**
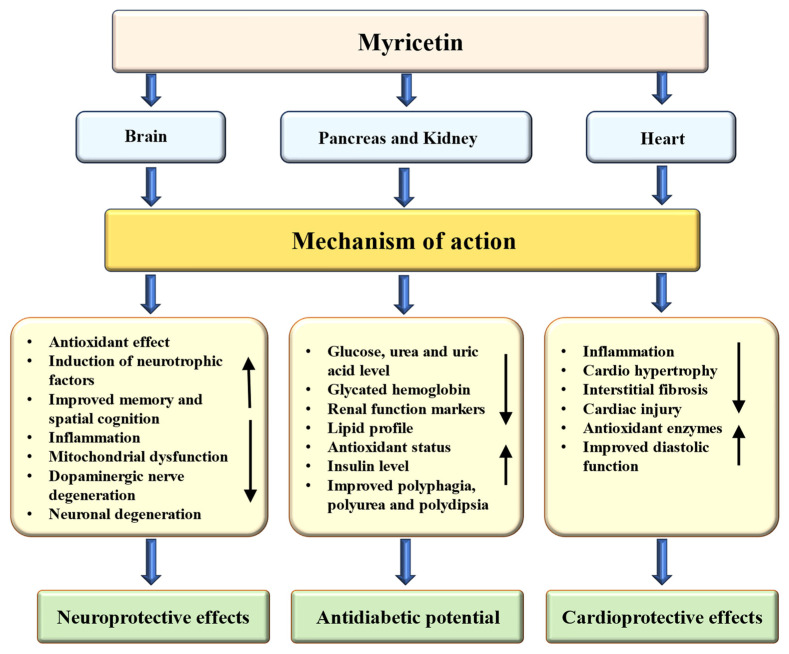
Anti-diabetic, neuroprotective and cardioprotective potential of myricetin through various mechanisms. The downward-pointing arrow shows downregulation, whereas the upward arrow represents upregulation.

Fruits, leaves, seeds, bark, and peels are frequently acknowledged as rich sources of bioactive phytochemicals that may aid in addressing various health concerns [85,86]. A range of natural compounds and their bioactive components have been shown to have anti-diabetic effects by decreasing inflammation and oxidative stress while preserving the structure of liver and kidney tissues [87,88,89]. The role of myricetin in the management of diabetes and its associated complications is summarized in Table 2. Myricetin’s role as an anti-diabetic through the modulation of different mechanisms is presented in Figure 3.

The role of myricetin was examined by enzymes of carbohydrate metabolism and renal function markers in streptozotocin (STZ)-cadmium (Cd) induced diabetic nephrotoxic animals. The innovative finding was noted as a substantial rise of plasma glucose, urea, uric acid, hemoglobin, and creatinine, and a substantial decrease of plasma insulin, hexokinase, hemoglobin, and glycogen were noticed in the STZ-Cd-induced diabetic rats. The administration of myricetin meaningfully normalizes the carbohydrate metabolic products like gluconeogenic enzymes, glycated hemoglobin, glucose, and glycogen phosphorylase, and renal function markers with increased insulin, glycogen, and glycogen synthase [90]. Diabetic nephrotoxic rats exhibited meaningfully elevated levels of urinary albumin along with altered lipid profiles. Alongside this, there was a decrease in the activities of lecithin cholesterol acyl transferase and lipoprotein lipase. Treatment with myricetin was found to normalize these parameters. Furthermore, the administration of myricetin led to a decrease in interstitial fibrosis glomerulosclerosis and an expansion of the extracellular mesangial matrix in diabetic nephrotoxic rats. Also, myricetin confirmed considerable protective effects against the lipid metabolism alterations induced by STZ-Cd, thereby mitigating diabetic nephropathy in the experimental rats [91].

To prove the role of myricetin in improving the symptoms of type 2 diabetes, as well as controlling the intestinal flora in a type 2 diabetes mouse model, Iit was noticed that fasting blood glucose and blood lipid levels of Type 2 diabetes mellitus (T2DM) mice were significantly decreased by myricetin treatment, whereas there were increased SOD levels. Myricetin improved polydipsia, polyuria, polyphagia and weight loss in T2DM mice [92]. It was reported that myricetin reduced diabetic cardiomyopathy-associated cardiac damage in mice subjected to streptozotocin and in neonatal rat cardiomyocytes challenged with high glucose. Myricetin treatment meaningfully alleviated cardiac hypertrophy and interstitial fibrosis apoptosis mechanically. Myricetin treatment increased the activity of the Nrf2/HO-1 pathway, strengthening antioxidative stress capacity and decreasing MDA production. These beneficial effects of myricetin treatment protected cardiomyocytes from apoptosis [93]. Myricetin alleviated renal dysfunction, oxidative damage, and fibrosis induced by diabetes mellitus while enhancing the expression of Nrf2 and its target genes. After Nrf2 was knocked down, myricetin treatment still significantly reduced diabetes-induced renal dysfunction and fibrosis. It was concluded that myricetin prevented the diabetes-associated decrease in expression of Nrf2 and the IκB/NF-κB (P65) signaling pathway was inhibited [94].

A recent finding reported that myricetin improves cardiac function in diabetic cardiomyopathy mice by decreasing interstitial fibrosis and cardiomyocyte hypertrophy. Moreover, myricetin caused an increase in occludin expression and the number of goblet cells in diabetic cardiomyopathy mice. Compared with diabetic cardiomyopathy mice unfed with gut content, the expression of occludin, the cardiac function, and the number of goblet cells in diabetic cardiomyopathy mice fed by gut contents were higher, whereas TLR4/MyD88 pathway-related proteins and cardiomyocyte hypertrophy were reduced [95]. The mechanism of myricetin, on a high-fat diet (HFD) fed streptozotocin (STZ) induced diabetic rats was investigated. The results exhibited that administration of myricetin in HFD/STZ-induced diabetic rats dose-dependently reduced the serum glucose as well as insulin. Moreover, myricetin protected pancreatic tissue from HFD-fed STZ-induced apoptosis. The experimental results indicate that myricetin offers noteworthy health benefits and could be measured an auspicious dietary complement for supporting treatment of hypoglycemic [96].

A study was made to reconnoiter the role of myricetin on insulin resistance in rats fed with a diet containing fructose. Frequent intravenous injection of myricetin was found to reduce the high triglyceride and glucose levels. Moreover, the higher degree of insulin resistance in fructose-chow-fed rats was diminished by myricetin treatment. Also, treatment with myricetin reversed the decreased insulin action on the phosphorylation of insulin receptors, insulin receptor substrate 1, and Akt in the soleus muscle of rats [97]. Diabetic nephrotoxic rats exhibited a significant increase in the activities of hepatic and renal functional markers in their plasma. This was accompanied by an increase in albumin levels and urine volume. After 30 days of treatment with intraperitoneal administration of myricetin, the diabetic nephrotoxic rats showed significant protective effects on all the biochemical parameters examined. The results indicate that myricetin at a 1.0 mg/kg body weight dose demonstrates a greater antihyperglycemic and renoprotective effect [98]. Filiz Ozcan et al. (2012) reported that myricetin treatment reduced blood urea nitrogen (BUN) and urinary volume, decreased glomerulosclerosis, and decreased protein excretion, which was intensely increased in diabetic rats. Reduced creatinine clearance measured in diabetic rats was increased after treatment of myricetin. The study revealed that myricetin restored renal activities of glutathione peroxidase (GPx) and xanthine oxidase (XO), and improved altered renal functions in diabetic rats [99]. Myricetin, administered at an effective dose of 1.0 mg/kg, decreased the rise in plasma glucose levels. Additionally, myricetin demonstrated a concentration-dependent stimulatory effect on glucose uptake in the soleus muscles isolated from STZ-diabetic rats [100].

**Table 2:** Anti-diabetic activities of myricetin through different mechanisms.

Activity	Types of Study	Doses	Findings	Ref.
Anti-diabetic potential	Male albino Wistar rats, in vivo	1.0 mg/kg bw	○ Myricetin normalizes the carbohydrate metabolic products ○ Histological changes of the kidney, liver and pancreas tissues protected	[90]
	Male albino Wistar rats, in vivo	1.0 mg/kg bw	○ Myricetin regulates the glucose hemostasis, regulating lipid metabolism ○ It maintained changes in lipid metabolism	[91]
	Male mice, in vivo	75, 150 and 300 mg/kg	○ Myricetin improved lipid content, SOD, FBG ○ Myricetin normalized the intestinal flora	[92]
	Mice model, in vivo	200 mg/kg/day	○ This compound alleviated cardiac hypertrophy and interstitial fibrosis and decreased MDA production	[93]
	Mice model, in vivo	100 mg/kg/day	○ Myricetin alleviated renal dysfunction, and oxidative damage and fibrosis	[94]
	Rat model, in vivo	50 and 200 mg/kg body weight	○ Myricetin reduced the serum glucose as well as insulin ○ Myricetin enhanced the expression of insulin receptor	[96]
	Rats model, in vivo	1 mg/kg per injection	○ Myricetin enhances insulin sensitivity	[97]
	Rats model, in vivo	0.5, 1.0 and 1.5 mg/kg bw	○ Myricetin acts as an antihyperglycemic and protects against kidney injury	[98]
	Rats model, in vivo	6 mg/day	○ Myricetin improved impaired renal functions and restored antioxidant enzyme activities	[99]
	Rats model, in vivo	1.0 mg/kg	○ Myricetin decreased the rise in plasma glucose levels	[100]

### 4.5. Cardioprotective Effects

Cardiovascular diseases (CVDs) rank among the top causes of illness and death worldwide, with increasing incidence rates and declining age of onset in recent years [101]. Various biochemical, genetic, environmental, as well as behavioral factors can play a role in the emergence of cardiovascular diseases [102]. As far back as the 1990s, epidemiological studies showed a link between higher consumption of flavonoid-rich diets and a reduced incidence of cardiovascular disease [103]. The role of myricetin in the management of cardio-associated pathogenesis is summarized in Table 3. Myricetin’s role as cardioprotective through the modulation of different mechanisms is presented in Figure 3.

Recently, numerous studies have highlighted the preventive and therapeutic benefits of flavonoids for the cardiovascular system, both on their own and in combination with other agents [104]. Lipopolysaccharide significantly impaired the mouse cardiac function, but myricetin administration markedly improved it. Myricetin also substantially decreased the production of inflammatory cytokines in both serum and cardiac tissue. Myricetin has been shown to inhibit the degradation of IκBα, nuclear translocation of p65, and cellular apoptosis, both in vivo and in vitro [62]. Pretreatment with myricetin (100 mg/kg and 300 mg/kg, both administered orally) for 21 days significantly reduced the effects of isoproterenol on heart rate, as well as levels of AST, LDH, CK, and SOD, and CAT [105]. A study found that myricetin significantly reduced the overexpression of IL-1beta, IL-6, and TNF-alpha by inhibiting the NF-κB/P65 signaling pathway. Additionally, treatment with myricetin resulted in lower levels of reactive oxygen species (ROS) and increased the expression of superoxide dismutase and glutathione peroxidase [106]. In comparison to the I/R group, pretreatment with 5μM myricetin improved several cardiovascular parameters. This treatment enhanced the maximum up/down rate of left ventricular pressure (dp/dt_max_) and coronary flow, increased left ventricular developed pressure, and reduced levels of creatine kinase. Additionally, myricetin was found to have beneficial effects by decreasing both infarct size and cardiomyocyte apoptosis. It also demonstrated antioxidant properties, evidenced by a reduction in malondialdehyde (MDA) levels, alongside an increase in superoxide dismutase (SOD) levels and an improved glutathione (GSH)/glutathione disulfide (GSSG) ratio [107]. In vivo studies showed that myricetin treatment alleviated cardiac hypertrophy, apoptosis, and interstitial fibrosis. Mechanistically, myricetin significantly enhanced the activity of the Nrf2/HO-1 pathway, which improved oxidative stress resistance. This was evidenced by increased activity of GPx and SOD, alongside decreased production of MDA. The protective effects of myricetin treatment on cardiomyocytes were demonstrated by a reduction in terminal deoxynucleotidyl transferase dUTP nick end labeling (TUNEL)-positive nuclei, c-caspase 3, and Bax, indicating decreased apoptosis [93]. In vitro, studies demonstrated that pretreatment with myricetin notably lowered the expression of inflammatory cytokines triggered by advanced glycation end-products (AGEs), while also reducing cell apoptosis, fibrosis, and hypertrophy in H9c2 cells. In vivo investigations demonstrated that oral administration of myricetin markedly lowered the expression of enzymes linked to cardiomyopathy. Additionally, myricetin improved diastolic dysfunction and alleviated histological changes [108]. Myricetin may enhance cardiac function in mice with diabetic cardiomyopathy by reducing cardiomyocyte hypertrophy and interstitial fibrosis. It has been shown to increase the expression of occludin and the number of goblet cells in these mice. When comparing diabetic cardiomyopathy (DCM) mice that were not fed gut content with those that were, the latter demonstrated improved cardiac function, increased goblet cells, and higher occludin expression. Conversely, the DCM mice that were fed gut content exhibited a decrease in cardiomyocyte hypertrophy and in the levels of proteins related to the TLR4/MyD88 pathway [95].

Myricetin reduced cardiac injury caused by doxorubicin, leading to decreased levels of cardiac troponin I (cTnI), AST, lactate dehydrogenase (LDH), and brain natriuretic peptide (BNP). It also improved myocardial injury and fibrosis. Furthermore, myricetin effectively prevented doxorubicin-induced oxidative stress by increasing the activities of GSH, SOD, CAT, and lowered MDA levels [109]. After 21 days of treatment, it was observed that myricetin significantly reduced myocardial injury in mice with experimental autoimmune myocarditis and the treatment led to a decrease in serum levels of anti-cardiac myosin antibodies [110]. A study was planned to explore the effects of myricetin against 5-FU-caused cardiac injury in rats. It was noticed that 5-FU injection caused inflammation, extensive cardiac damage and oxidative stress. However, myricetin lessened markers of inflammation, oxidative stress, apoptosis, and cardiac toxicity [111]. Myricetin exposure caused dose-dependent suppression of PGN-induced COX-2 expression and reduced IκB-α degradation, consistent with decreased COX-2 activity. The above-mentioned results advise that myricetin is useful for regulating the inflammatory response in infective endocarditis [60].

**Table 3:** Cardioprotective activities of myricetin through different mechanisms.

Activity	Model	Dose	Outcome	Ref.
	Mice, in vivo	100 mg/kg	○ Myricetin caused a decrease in inflammatory cytokines ○ Myricetin attenuated LPS induced myocardial dysfunction	[62]
	Wistar rats, in vivo	100 and 300 mg/kg, p.o	○ Pretreatment with myricetin reduced the effects of isoproterenol on heart rate ○ Additionally, myricetin exhibited a lesser degree of histopathological changes	[105]
	H9c2 cardiomyocyte cell line, in vitro	5, 10, 20, 40 Μm	○ Myricetin blocked the production of inflammatory marker	[106]
	Rat model, in vivo	5 μM	○ Myricetin has protective effects against myocardial injury ○ Myricetin reduces levels of cardiomyocyte apoptosis	[107]
	Mice model, in vivo	200 mg/kg/d	○ Myricetin caused protective effects via enhancing Nrf2/HO-1 and inhibiting I*κ*B*α*/NF*κ*B	[93]
	H9c2 cells, in vitro	25 μg/mL	○ Incubation with AGEs in H9c2 cells promoted TNF-α and phosphor-IKK- expression and myricetin reduced alterations	[108]
	Mice model, in vivo	300 mg/kg/day	○ Myricetin reduced the expression of enzymes related to cardiomyopathy and inflammatory cytokines	[108]
	Male rats, in vivo	2.5 mg/kg and 5 mg/kg	○ Myricitrin prevented the Dox-induced cardiac injury	[109]
	Rats model, in vivo	25 and 50 mg/kg	○ Myricetin alleviates 5-FU-caused cardiac damage via modulating and oxidative stress inflammation	[111]
	H9c2 cells, in vitro	1, 5, 10, & 15 μM	○ Myricetin inhibits PGN-induced IκB-α degradation	[60]

### 4.6. Neuroprotective Effects

Neurodegenerative diseases are a heterogeneous group of disorders considered by progressive damage as well as loss of neurons in different areas of the central or peripheral nervous system [112]. Neurodegenerative disorders such as Parkinson’s disease, Alzheimer’s disease, and multiple sclerosis affect millions of people worldwide, resulting in substantial socio-economic challenges [113]. There are several medications available for treating this pathogenesis; however, these drugs may lead to undesirable side effects. However, safe alternative medicines are needed to overcome these adverse effects and play a role in preventing this condition. The role of myricetin in the management of neuro-associated pathogenesis is summarized in Table 4. Myricetin’s role as neuroprotective through the modulation of different mechanisms is presented in Figure 3.

Matin Ramezani et al., 2016, investigated the potential effects of myricetin and it significantly improved learning and memory impairments and increased the number of hippocampal CA3 pyramidal neurons in rats with Alzheimer’s disease [114].

Myricetin treatment improved learning memory and ameliorated tau phosphorylation and reduced pre- and postsynaptic proteins in Aβ_42_ oligomer-treated neuronal SH-SY5Y cells and 3 × Tg mice [115]. The effect of myricetin on Alzheimer’s disease (AD) and its causal mechanisms were studied. Myricetin efficiently reduced Fe^2+^-induced cell death in SH-SY5Y cells in vitro. In a mouse model of AD, myricetin treatment meaningfully reversed scopolamine-induced cognitive deficits and down-regulating brain iron. Consequently, it was suggested that myricetin treatment reduced mice’s cognitive deficits by preventing AChE and brain iron regulation [116]. The Parkinson’s disease (PD) models were made by treating SH-SY5Y cells with 1-methyl-4-phenylpyridinium (MPP^+^) and injecting 1-methyl-4-phenyl-1,2,3,6-tetrahydropyridine (MPTP) into rats, correspondingly. The results confirmed that myricetin treatment efficiently mitigated MPTP-triggered motor impairment, α-synuclein (α-Syn) accumulation, and dopamine neuronal death in PD models. In vitro, myricetin treatment restored SH-SY5Y cell viability as well as alleviated MPP^+^-induced SH-SY5Y cell ferroptosis [117]. The role of myricetin in Parkinson’s disease models based on in vivo and in vitro experiments was explored. Myricetin treatment suppressed the expression of pro-inflammatory mediators, the activation of microglia, and the reduction in the number of dopaminergic neurons, as well as ameliorated the rats’ motor dysfunction. Also, myricetin inhibited the activation of the mitogen-activated protein kinase (MAPK) and NF-κB pathways and the production of pro-inflammatory mediators in activated microglia. Based on these results, myricetin inhibits dopaminergic neuron degeneration via inhibiting microglial neuroinflammation [118]. Rotenone administration caused dopaminergic neuronal degeneration, memory decline, impaired muscular coordination, dopamine depletion, gait disturbances, oxidative stress, and apoptosis. The ingestion of myricetin by *Drosophila* meaningfully prevented neuronal degeneration caused by rotenone [119]. Myricetin demonstrated a dose-dependent increase in antioxidative activity. When PD flies were exposed to various concentrations of myricetin, there was a significant dose-dependent rise in dopamine levels compared to unexposed PD flies. Additionally, myricetin helped protect against the loss of dopaminergic neurons in the brains of the PD flies [120]. The effects of myricetin on 6-hydroxydopamine (6-OHDA)-induced neurodegeneration was tested. The dopamine content in the striatum was reduced after 6-OHDA treatment, which was restored by myricetin treatment. Moreover, it showed that myricetin prevented the 6 -OHDA-induced decrease of tyrosine hydroxylase positive neurons [121]. The role of the myricetin, in a pentylenetetrazole (PTZ)-induced mouse model of epilepsy was examined. Myricetin treatment reduced the mortality rate and seizures. Elevated expression levels of apoptotic proteins and increased apoptotic cell count caused by PTZ were downregulated following myricetin treatment. The results of the study showed that myricetin may cause protective effects via regulating the molecular events associated with epileptogenesis [122]. It significantly enhanced the activities of antioxidant enzymes and lowered markers of oxidative stress. Myricetin also effectively decreased the levels of pro-inflammatory cytokines, showing its anti-inflammatory properties. Behavioral evaluations indicated that myricetin improved motor functions and cognitive abilities in PTZ-treated mice, leading to a notable reduction in mortality rates and severity of seizures [123].

**Table 4:** Neuroprotective activities of myricetin through different mechanisms.

Disease	Types of Study	Model	Doses	Outcome	Ref.
Alzheimer’s disease	In vivo	Rat models	5 or 10 mg/kg	○ Myricetin improved learning and memory impairments	[114]
	In vivo	Mice model	20 mg/kg	○ Myricetin improved spatial cognition and memory	[115]
	In vitro	SH-SY5Y cells	5–20 µM	○ Myricetin ameliorated Aβ_42_O-induced synaptic impairment	[115]
	In vitro	SH-SY5Y	4, 1, 0.25, 0.063, 0.016 µM	○ Myricetin meaningfully increased the cell viability in a concentration-dependent way	[116]
	In vivo	Mice model	25 or 50 mg/kg	○ Myricetin reduced cognitive deficits in mice through AChE as well as brain iron regulation inhibition ○ Myricetin decreased iron contents through transferrin receptor 1 expression inhibition	[116]
Parkinson’s disease	In vitro	SH-SY5Y cells	50 µM	○ Myricetin suppressed cell ferroptosis and promoted the nuclear translocation of Nrf2	[117]
	In vivo	Rat model	25 g/kg	○ Myricetin mitigated motor impairment ○ Myricetin decreased dopamine neuronal death	[117]
	In vivo	Rat model	2.5, 5, or 10 mg/kg	○ Myricetin treatment ameliorated the behavioral dysfunction and alleviates activation of microglia	[118]
	In vitro	SH-SY5Y cells	12.5, 25, 50 µM	○ Myricetin suppressed neurotoxicity and reduced inflammatory responses	[118]
	In vivo	Drosophila model	250, 500, 750, 1000 µM	○ Ingestion of myricetin by *Drosophila* prevented neuronal degeneration	[119]
	In vivo	Drosophila Model	10, 20 and 40 μM	○ Myricetin increased in antioxidative activity and dopamine content	[120]
Epilepsy	In vivo	Mice model	50, 100 mg/kg	○ Myricetin reduced seizure and mortality rates ○ Elevated expression levels of apoptotic proteins downregulated by myricetin treatment	[122]
	In vivo	Mice model	200 mg/kg	○ Myricetin decreased pro-inflammatory cytokines and improved cognitive and motor functions	[123]

### 4.7. Anti-Cancer Potential

**Figure 4:**
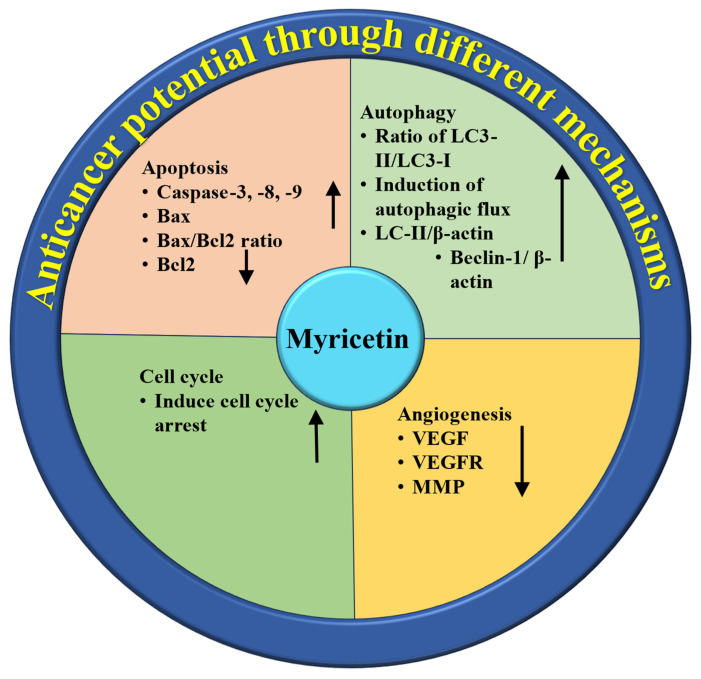
Anti-cancerous effects of myricetin via modulation of cell signaling molecules. The downward-pointing arrow shows downregulation, whereas the upward arrow represents upregulation.

Cancer poses a severe threat to both physical and mental health, with high incidence and mortality rates globally [124,125]. Presently, the leading treatments for cancer consist of radiotherapy, chemotherapy, and surgical procedures. Despite their effectiveness, these approaches have limited efficacy and may also lead to negative side effects. Ongoing research and development efforts are focused on discovering new cytotoxic compounds from medicinal plants that exhibit antiproliferative activity [126] and in vitro studies have shown the activities of flavonoids on tumor cells, such as the inhibition of cell growth and alteration of tumor invasive behavior [127]. Various medicinal plants and their bioactive compounds, including myricetin, demonstrate anti-cancer potential both in vivo and in vitro by modulating cell signaling pathways. Natural compounds and their bioactive compound role in cancer management have been established through their ability to target several molecules and signaling pathways, including those involved in angiogenesis, cell cycle, apoptosis, autophagy, tumor suppressor genes, and inflammation [128,129,130]. Myricetin’s role as an anti-cancer through the modulation of different mechanisms is presented in Figure 4. Moreover, the role of myricetin in the management of cancer is summarized in Table 5. A study focused on ovarian cancer found that myricetin inhibited the secretion of the angiogenesis mediator vascular endothelial growth factor (VEGF) and reduced the levels of p-Akt and hypoxia-inducible factor-1α (HIF-1α) proteins in ovarian cancer cells. Additionally, an innovative pathway involving p21/HIF-1α/VEGF was involved in myricetin’s repressing effect on angiogenesis in cancer cells [131]. Another study based on both in vivo and in vitro experiments revealed that myricetin reduced vascular endothelial growth factor levels. Additionally, Western blot analysis indicated that myricetin downregulated the expression of p38MAPK and VEGFR2 [132].

The role of myricetin in the induction of ferroptosis in breast cancer cells was explored. It was noticed that myricetin could inhibit 4 T1 tumor cell viability and colony-forming activity, increasing the level of ROS, MDA, Fe^2+^, and within these cells. From a mechanistic perspective, this bioactive compound induced ferroptotic 4 T1 cell death by downregulating glutathione peroxidase 4 (GPX4) and Nrf-2. Moreover, in vivo, findings established that myricetin treatment was enough to decrease the growth of subcutaneous breast tumors in female mice, as noticed by decreases in tumor volume and weight [133]. A study was conducted to evaluate the role of myricetin in inducing apoptosis in gastric cancer cells. It was found that myricetin decreased the survival rate of these cells by inhibiting the phosphoinositide 3 kinase (PI3K)/Akt/mammalian (or mechanistic) target of rapamycin (mTOR) pathway, thereby triggering apoptosis. Similar findings were observed in vivo, where tumor growth was also suppressed [134]. A study on gastric cancer indicated that the percentage of apoptotic cells was higher in the treatment groups compared to the control groups. Additionally, the levels of the anti-apoptotic protein Bcl-2 and pro-caspase-3 were reduced in the myricetin treatment group. In contrast, the pro-apoptotic protein Bax and cleaved caspase-3 were meaningfully elevated in the myricetin-treated groups [135].

A study based on pancreatic cancer reported that the presence of myricetin considerably reduced cell viability in all pancreatic cancer cells tested (S2-013MIA and PaCa-2, Panc-1,) in a dose-dependent behavior, while little effect was detected on the viability of normal pancreatic ductal cells; it was noticed that myricetin-induced cell death in pancreatic cancer cells is facilitated by apoptosis. The incubation of S2-013 and MIA PaCa-2 cells by myricetin inhibited the phosphorylation of Akt and results indicate that myricetin causes apoptosis through the inhibition of the PI-3 kinase signaling pathway. Furthermore, myricetin inhibits tumor growth in an orthotopic mouse model of pancreatic cancer [136]. Myricetin employs anticancer effects in human glioma cells by inducing mitochondrial-mediated apoptosis, ROS generation, G2/M phase cell cycle arrest, and cell migration inhibition [137]. Myricetin promotes autophagy and halts the cell cycle at the G2/M phase, which helps inhibit the growth of HCC cells by lowering MARCH 1 levels. In both Hep3B and HepG2 cells, myricetin decreases the membrane-associated RING-CH finger protein 1 (MARCH1) protein. Additionally, myricetin suppresses HCC growth both in vitro and in vivo by inhibiting the p38 MAPK and Stat3 signaling pathways through the downregulation of MARCH 1 [138]. Another study result based on MTT assay proved that exposure of HepG2 cells to myricetin activated G2/M phase arrest. Myricetin evidently diminished Cdc2 and cyclin B1 protein levels and increased the protein levels of the p53/p21 cascade in HepG2 cells. Furthermore, treatment by myricetin caused in the Thr14/Tyr15 phosphorylated (inactive) p27 and Cdc2 up-regulation, and the downregulation of cyclin-dependent kinase 7 (CDK7) kinase protein, as well as CDK7-facilitated Thr161 phosphorylated (active) Cdc2 [139].

### 4.8. Role in Respiratory Disease

**Figure 5:**
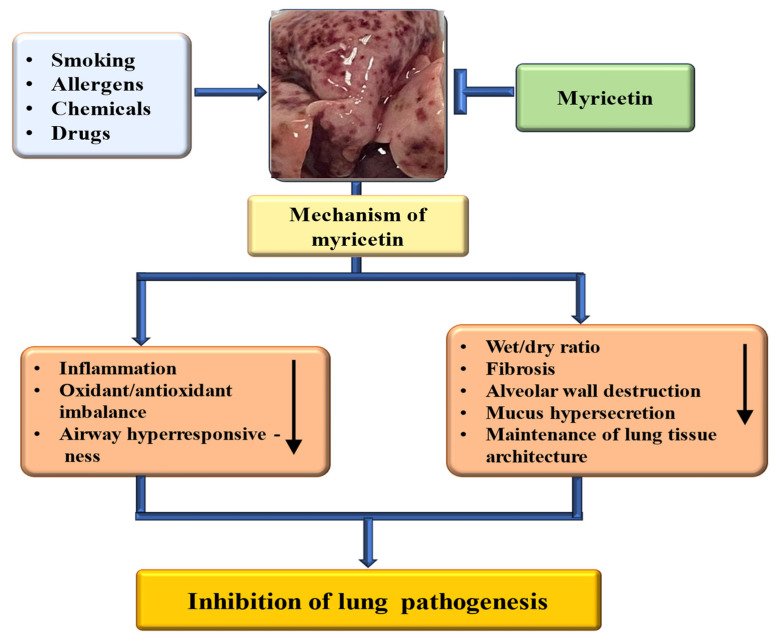
The role of myricetin in managing lung pathogenesis through different mechanisms (the lung image used in the figure is from our lab). The downward-pointing arrow shows downregulation.

The rising incidence of chronic respiratory diseases (CRDs) has led to higher rates of illness and death globally [143]. The existing treatment approach is costly and can lead to adverse side effects. Therefore, there is a need for safer and more affordable treatment options to tackle this pathogenesis. Natural compounds and their bioactive have a proven role in managing lung-associated pathogenesis [144,145]. Myricetin’s role in lung pathogenesis through the modulation of the different mechanisms is presented in Figure 5. Moreover, the role of myricetin in the management of respiratory system-associated pathogenesis is summarized in Table 6. The murine sepsis model was made by cecal ligation and puncture (CLP), and the role of myricetin in this pathogenesis was examined. The survival rate test designated that myricetin suggestively improved the vitality of CLP-operated mice. Moreover, myricetin showed significant inhibitory effects on pathological changes in morphology, oxidative stress response biomarkers of inflammatory response, and mitochondrial damage in CLP-induced mice [146]. Myricetin reduced lung inflammation, as evidenced by the reduced wet-to-dry weight ratio of lungs, MPO activity, and protein concentration in the BALF, inflammatory cell migration, and cytokine production. A decrease was also seen in TLR4, NF-κB, and MyD88 expressions. Moreover, an elevated antioxidant enzyme activity of catalase, superoxide dismutase, and glutathione peroxidase was observed in all the treatment groups [67]. The results of another study reported that compared with the model group, the levels of neutrophils and macrophages in BALF in the myricetin and active groups decreased, whereas the wet/dry ratio of the lung tissue and total protein decreased [147].

A study was performed to inspect whether myricetin reduced airway hyperresponsiveness, eosinophil infiltration, and airway inflammation in the lungs of asthmatic mice. It was reported that myricetin efficiently mitigated eosinophil infiltration, goblet cell hyperplasia, and airway hyperresponsiveness (AHR) in the lungs, and it reduced the expression of Th2 cytokine in BALF from asthmatic mice. Myricetin reduced ROS and the production of proinflammatory cytokines, eotaxins, in BEAS-2B cells [148]. The in vivo studies showed that myricetin effectively alleviated bleomycin (BLM)-induced pulmonary fibrosis. In vitro studies designated that myricetin dose-dependently suppresses TGF-β1/Smad signaling and reduces epithelial-mesenchymal transition and TGF-β1-induced fibroblast activation [149].

### 4.9. Effects of Myricetin on Digestive System/Inflammatory Bowel Disease

Inflammatory bowel disease (IBD) is characterized by recurrent episodes of gastrointestinal inflammation caused by an atypical immune response to the intestinal microbiota [150]. Inflammatory bowel disease chiefly involves two distinct clinical phenotypes: Crohn’s disease (CD) and ulcerative colitis (UC) [151]. Since the year 2000, the global incidence of IBDs has been on the rise, now impacting approximately 1 in 200 people in Western nations [152]. Myricetin’s role in the digestive system and associated pathogenesis is presented in Table 6. The protective potential of myricetin was assessed in a murine model of colitis induced by dextran sulphate sodium. The results demonstrated that myricetin treatment reduced histology scores and ameliorated body weight loss in a dose-dependent means. Myricetin diminished myeloperoxidase production while increasing the activity of glutathione peroxidase and superoxide dismutase. Additionally, the levels of the cytokine’s interleukin-6 interleukin-1 decreased [153]. The M10 derivative of myricetin, administered orally, demonstrated a reduction in ulcerative colitis, significantly lowering the disease activity index. Pathological examination indicated that M10 alleviated the extent of colonic inflammation in the affected tissues and helped restore the integrity of the intestinal barrier that had been compromised by Dextran sulphate sodium (DSS) [154]. Myricetin ameliorated the severity of inflammation in acute ulcerative colitis and meaningfully improved the condition. Myricetin elevated the levels of transforming growth factor β and IL-10. Furthermore, the proportion of regulatory T cells increased in mice in the treatment of the myricetin group [155]. M10, a derivative of myricetin, enhanced the populations of CD8+ T and CD4+ T cells while inhibiting the infiltration of myeloid-derived suppressor cells into colorectal tissues. Additionally, there was a decrease in pro-inflammatory mediators, including granulocyte-macrophage colony-stimulating factor/macrophage colony-stimulating factor, IL-6, and TNF-α within the colonic mucosa [156].

### 4.10. Anti-Obesity Properties

**Figure 6:**
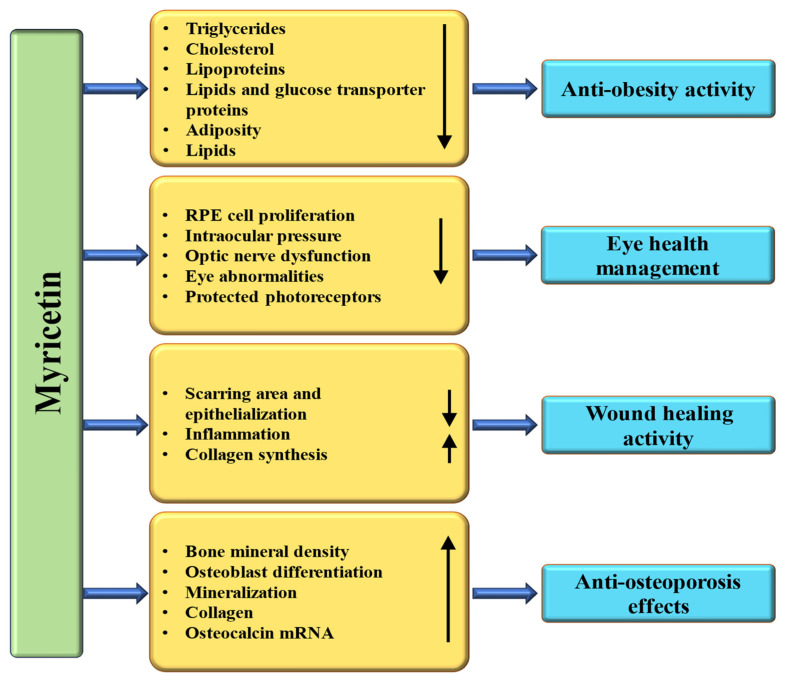
Role of myricetin in eye disease, bone disease, obesity, and wound healing. The downward-pointing arrow shows downregulation, whereas the upward arrow represents upregulation.

Obesity is a substantial worldwide public health issue, described as having a Body Mass Index of 30 kg/m^2^ or higher [157]. Individuals who are obese face an increased risk of developing various chronic diseases. Bioactive compounds are present in small amounts in these food items, and they are effective in treating obesity [158,159]. Myricetin’s role in anti-obesity through modulation of different mechanisms is presented in Figure 6. Moreover, the role of myricetin in anti-obesity is summarized in Table 6. A study was performed to examine myricetin’s role in reducing mice’s body weight induced by a high-fat diet (HFD). Administration of myricetin intensely decreased the body weight of diet-induced obese mice. Numerous parameters linked to obesity, including serum triglyceride, glucose, and cholesterol, were reduced in myricetin-treated mice. Furthermore, obesity-linked oxidative stress was ameliorated in myricetin-treated mice [49]. A study finding revealed that myricetin inhibited the differentiation of 3 T3-L1 preadipocytes in a concentration-dependent way. It was observed that myricetin lowered both the mRNA and protein levels of key adipogenic transcription factors. Additionally, myricetin treatment resulted in a reduction of mRNA levels for several other transcription factors related to adipogenesis. These results indicate that myricetin exhibits anti-obesity effects in adipocytes [160].

After 14 weeks of myricetin treatment, db/db mice showed improvements in systemic insulin resistance, hepatic steatosis, and a reduction in body weight. Myricetin also improved plasma lipid profiles, increased energy expenditure, and reduced adiposity. In inguinal white adipose tissue (Iwat), treatment with myricetin prompted the formation of beige adipocytes, activated mitochondrial biogenesis, and elevated the expression of thermogenic proteins. Moreover, the expression levels of adiponectin were elevated in C3H10T1/2 cells and in adipose tissues and plasma following myricetin treatment [161]. Research indicated that myricetin decreased the intracellular buildup of triglycerides in 3T3-L1 adipocytes from rats on a high-fat diet. Its impact on reducing body weight and visceral fat pad weights in fat-fed rats was comparable to that of fenofibrate administered at a dose of 100 mg/kg/day [162].

### 4.11. Wound Healing Effects

Numerous studies have investigated the wound-healing abilities of natural products that exhibit antioxidant, collagen-promoting, anti-inflammatory, and antibacterial properties [163]. The role of myricetin in wound healing through modulation of different mechanisms are presented in Figure 6. Moreover, the wound-healing role of myricetin is summarized in Table 6. A topical application of naturally isolated myricetin from the shoots of *Tecomaria capensis* v. *aurea* on wound healing was made in albino rats. It was reported that the percentage of wound closure as well as contraction was delayed in wounded rats (67.35%) and was increased after treatment of wounded rats with myricetin; the treatment with myricetin (20%) was the most powerful (98.76%). Histological studies indicated that treatment with 10% myricetin resulted in a significant area of scarring at the wound site, similar to the control group. In contrast, the 20% myricetin treatment showed a reduced scarring area and re-epithelialization accompanied by a higher density of fibroblasts at the wound site. Therefore, it can be proposed that the enhancements in inflammatory cytokines as well as systemic reorganization after treatment of myricetin may be suggested to play a vital part in the promotion of wound healing [164]. Results presented that the topical application of myricetin-3-O-rhamnoside decreased inflammatory cells infiltration and increased wound healing [165]. An in vitro model of inflammation was created using monolayers of scratched fibroblasts or keratinocytes that were exposed to LPS from Pseudomonas aeruginosa. Subsequently, myricetin and dihydromyricetin were administered to the cells at sub-toxic concentrations ranging from 5 to 15 µM. It was reported that myricetin and dihydromyricetin inhibit the production of pro-inflammatory cytokines in LPS-stimulated skin cells and level of MMP-1 decreased in fibroblasts [166].

### 4.12. Anti-Analgesic Activity

The analgesic activity of myricetin, a chief compound in *Myrica rubra* Sieb. et Zucc. leaves were examined. It was reported that myricetin displayed a noteworthy inhibition on chemical nociceptive models. Additionally, myricetin lowered the levels of prostaglandin E2 (PGE2) in the peritoneal fluid and inhibited platelet aggregation induced by collagen and arachidonic acid in vitro [167]. The role of myricetin as an anti-analgesic is presented in Table 6. The analgesic effect of myricetin in a neuropathic pain model was examined. In vivo, a single injection of myricetin decreased SNL-induced mechanical allodynia as well as thermal hyperalgesia. In vitro, I(Ca(V)) (depolarization from −80 to 0 mV) was decreased by low concentrations of myricetin [168]. The antinociceptive effects of myricitrin in models of overt nociception was examined. The nociception induced by bradykinin was stopped by prior treatment with myricitrin. The myricetin dose of 100 mg/kg caused a 57% decrease in cinnamaldehyde-induced nociception [169]. Myricetin exhibited a notable inhibition in chemical nociceptive models, including the acetic acid-induced writhing response. It also reduced the levels of PGE2 in the peritoneal fluid and decreased platelet aggregation triggered by collagen and arachidonic acid. These findings collectively indicate that myricetin has strong analgesic properties, which are associated with peripheral analgesia [167].

### 4.13. Anti-Platelet Aggregation Potential

Studies indicate that flavonoids and phenolic acids function as anti-platelet aggregation agents [170,171], and myricetin also contributes to this effect. The antagonistic potential of myricetin on platelet activating factor (PAF) was evaluated. The specific binding of [3H] PAF to rabbit platelet receptors was examined via radioligand binding assay (RLBA). It was reported that the specific binding inhibition potency of myricetin was noticed to be concentration dependent. The platelet-activating factor-induced reactions of rabbit platelet adhesion, as well as polymorphonuclear leukocytes’ inner free calcium concentration increase, were prevented by myricetin [172]. The inhibitory effects of myricetin on the aggregation as well as secretion of platelets were examined. The flavonoid prevented platelet aggregation and ATP release of rabbit platelets brought by arachidonic acid or collagen, and somewhat those by platelet-activating factor [173]. Myricetin triggered an increase in platelet adenosine 3′,5′-cyclic monophosphate (cyclic-AMP) levels through prostacyclin stimulation. The anti-aggregating effect was due to modification in platelet cyclic-AMP metabolism, which involved the inhibition of phosphodiesterase activity [174]. It was reported that myricetin presented an important inhibition on chemical nociceptive models, such as the licking time on the late phase in the formalin test and acetic acid-induced writhing response in a dose-dependent way. Furthermore, myricetin inhibited the amount of PGE2 in the peritoneal fluid, and platelet aggregation was caused by collagen and arachidonic acid in vitro [167]. A recent finding reported that polyphenol-rich extract decreased platelet activation as well as aggregation induced by different agonists. Myricetin employed effective antiplatelet effects and reduced the capacity of platelets to spread on collagen [175].

### 4.14. Effect on Bone Disease

Myricetin’s role in glucocorticoid-induced osteoporosis was made, and outcomes exhibited that myricetin might reduce dexamethasone-induced osteoporosis by enhancing osteogenic differentiation and matrix mineralization through the ERK signaling pathway [176]. Myricetin’s role in bone disease is presented in Table 6. The myricetin treatment group’s bone mineral density (BMD) increased more than that of the diabetic group. Furthermore, myricetin treatment intensely improves trabecular bone microarchitecture by increasing bone mass and diminishing trabecular separation and structure model index compared to the control group [177]. The research focused on the protective effects of myricetin against destruction in human gingival fibroblasts (HGF) subjected to inflammatory conditions induced by lipopolysaccharide (LPS). Additionally, the study explored the role of myricetin in inhibiting osteoclastogenesis in cells caused by receptor activator of NF-κB ligand (RANKL) RAW264.7. Myricetin prevented the formation of TRAP (+) multinucleated cells. It also suppressed the activation of p-38, ERK, and cSrc signaling pathways and the degradation of I(k)B induced by RANKL in the RAW264.7 cells. Myricetin reduced the mRNA expression of osteoclast-linked genes, including cFOS, cathepsin K, and tartrate-resistant acid phosphatase (TRAP) [178]. Study results reported that mineralization of hBMSCs were improved by myricetin treatment. Myricetin raised the mRNA expression of osteocalcin, alkaline phosphatase, and collagen type I [179]. Myricetin, whether administered in high or low doses, effectively prevented the resorption of alveolar bone and enhanced the height of the alveolar crest in a mouse model. It also inhibited osteoclast formation and bone resorption. Notably, the high dose of myricetin showed greater efficacy than the low dose. This research indicated that myricetin positively influenced alveolar bone resorption in the mouse periodontitis model, suggesting its potential as a treatment option for both periodontitis and osteoporosis [180]. The effect of various flavonoids on bone calcium content and osteoclastogenesis were examined to compare action of flavonoid on bone formation as well as bone resorption. Mouse bone marrow cells were cultured in the presence of parathyroid hormone (PTH). Culture with PTH caused a substantial increase in osteoclast-like cell formation. This increase was meaningfully inhibited in the presence of quercetin, myricetin isorhamnetin, kaempferol, or curcumin in the range of 10^−8^−10^–6^ M. Furthermore, culture with PTH caused a noteworthy decrease in diaphyseal calcium content. This decrease was completely inhibited in the presence of myricetin, quercetin, kaempferol, or isorhamnetin of 10^−6^ M [181].

### 4.15. Effect on Eye Disease

**Figure 7:**
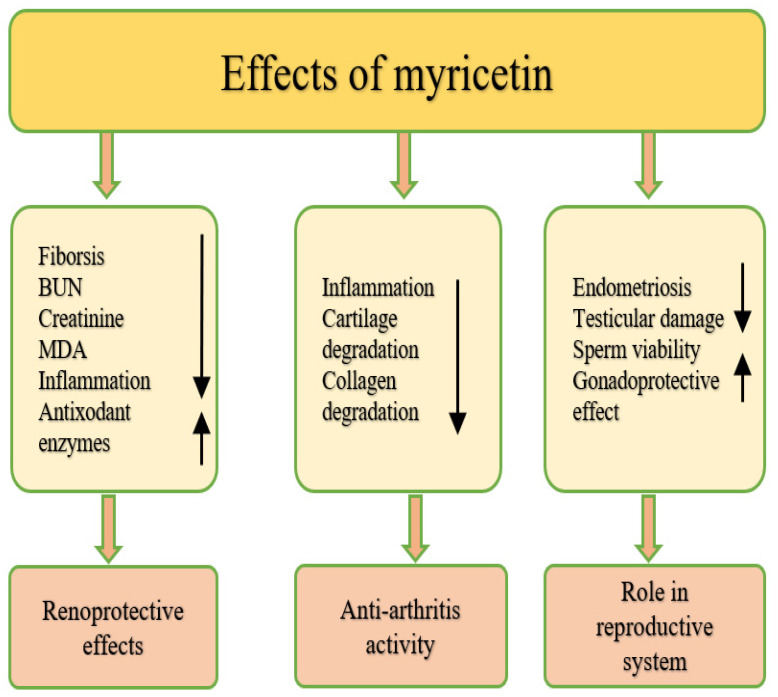
Role of myricetin as renoprotective, anti-arthritis, and in reproductive system. The downward-pointing arrow shows downregulation, whereas the upward arrow represents upregulation.

The impact of myricetin on the physiological characteristics and viability of cultured human retinal pigment epithelial (RPE) cells was examined. Myricetin was found to dose-dependently decrease RPE cell proliferation, VEGF secretion, and cell migration. Treatment with myricetin at low concentrations resulted in a reduction of VEGF gene expression, while higher concentrations led to an increase in its expression [182]. Myricetin’s role in wound healing through modulation of different mechanisms is presented in Figure 7. Moreover, the wound-healing role of myricetin is summarized in Table 6. The study was executed to check the intraocular pressure (IOP)-dropping activity of myricetin. It was reported that myricetin at doses of 1 mg reduced IOP to below control levels [183].

An important study examined the effect of myricetin on A2E and blue light-induced photoreceptor death in primary retinal cell cultures. Myricetin protected photoreceptors (100%) against blue-light-mediated damage. Myricetin also protected against A2E-induced photoreceptors and bipolar cell death. These results suggest that myricetin functions as a potent and effective neuroprotective agent for photoreceptor cells against A2E and light damage [184]. The role of myricetin on the trabecular meshwork cells in primary open-angle glaucoma (POAG) was examined. POAG TM cells exposed to myricetin showed lowered lipid peroxidation products and reactive oxidative species (ROS) levels. Myricetin effectively inhibited IOP elevation in glaucoma-induced rats and reduced inflammatory cytokines in the aqueous humor and POAG TM cells of glaucoma-induced rats [185]. The study examined whether gallic acid and myricetin-*rich Labisia pumila* extract (LP) consumption prevents diabetic eye disorders. The diabetic rats consuming LP revealed dose-dependent, histopathologically decreased eye abnormalities. The LP suppressed inflammation, vascular leakage, abnormal vascularization, oxidative tension, and hyperglycemia of the diabetic rats [186].

### 4.16. Effect on Osteoarthritis

Osteoarthritis (OA) is the most common progressive musculoskeletal condition that can affect joints, but it chiefly affects the knees and hips as predominant weight-bearing joints [187,188,189]. The currently used drugs are effective but also cause side effects. Myricetin has a confirmed role as an anti-arthritis agent through different mechanisms, including its anti-inflammatory potential. Myricetin inhibited IL-1β-induced production of PGE2 and nitric oxide (NO), expression of ADAMTS5, and degradation of collagen-II in mouse chondrocytes. Also, in vivo, myricetin decreased OA in the mouse model of osteoarthritis [190]. Myricetin reduced cartilage degeneration in vitro and in vivo. Moreover, myricetin decreased the IL-1β-induced inflammatory cytokines production. Myricetin-treated mice showed a less severe OA phenotype than vehicle-treated mice. More notably, myricetin intensely reduced the expression of chondrocyte catabolic and increased the secretion of chondrocyte anabolism agents, including proteoglycans and collagen [191]. Myricetin inhibited the generation of cytokines and inflammatory mediators and suppressed the production of cyclooxygenase-2, inducible nitric oxide synthase, and in human chondrocytes under IL-1β stimulation. Moreover, an in vivo study established that myricetin ameliorated the progression of osteoarthritis in mice [192].

### 4.17. Renoprotective Effects

The renoprotective role of myricetin was confirmed through different mechanisms. The myricetin nephroprotective role against cisplatin-caused nephrotoxicity was investigated. It was demonstrated that apigenin, myricetin, and their combination reduced serum creatinine (Cr), blood urea nitrogen (BUN), TNF-α, and MDA levels, and increased catalase and GSH levels parallel to histopathological improvement in kidney tissues. Myricetin showed a protective and hopefully a preventive strategy against nephrotoxicity due to their anti-inflammatory and antioxidant effects [66]. Myricetin’s renoprotective role through modulation of different mechanisms is presented in Figure 7. Moreover, the wound-healing role of myricetin is summarized in Table 6. The research investigated the nephroprotective properties of myricetin on the oxidant–antioxidant balance in the kidneys of mice that were given a high-fat diet along with ethanol. This combination elevated malondialdehyde levels in the kidneys, signifying oxidative stress and disrupting the antioxidant system. Myricetin significantly reduced renal oxidative damage by restoring malondialdehyde levels, increasing the activities of antioxidant enzymes, and rebalancing glutathione stores in the kidneys. These findings indicate that myricetin has nephroprotective effects by enhancing the renal antioxidative defense system [193]. The administration of myricetin was found to restore albumin and lipid profiles to normal levels. Histopathological examinations of kidney samples revealed that myricetin treatment suppressed extracellular mesangial matrix expansion, glomerulosclerosis, and interstitial fibrosis in diabetic nephrotoxic rats. Furthermore, results indicate that myricetin provided noteworthy protection against alterations in lipid metabolism induced by STZ-Cd, which reduced the incidence of diabetic nephropathy in experimental rats [91].

**Table 6:** The effects of myricetin in diverse pathogeneses via different mechanisms.

Activity	Study Types	Model	Dose	Outcomes	Ref.
Lung injury protective effects	In vivo	Mice model/murine sepsis model	100 mg/kg	○ Myricetin improved lung injury and survival rate ○ Myricetin alleviates inflammation and oxidative stress	[146]
	In vivo	Rat model	10, 20 and 40 mg/kg	○ Myricetin protects against lung injury ○ It inhibits NF-κB-mediated inflammatory responses	[67]
	In vivo	Mice model	50 mg/kg	○ Myricetin alleviates lung injury by reducing inflammatory reactions	[147]
Anti-ulcerative colitis effects	In vivo	Mice model	200, 100 or 50 mg/kg	○ Myricetin reduced histology scores ○ Myricetin reduced the production of inflammatory markers and myeloperoxidase	[153]
	In vivo	Mice model	80 mg/kg	○ Myricetin improved the inflammation severity	[155]
Anti-obesity potential	In vivo	Mice model	150 mg/kg	○ The administration of myricetin reduced cholesterol, glucose, and triglyceride levels ○ Obesity-associated oxidative stress inflammation ameliorated	[49]
	In vivo	Mice model	400 mg/kg	○ Myricetin prevents obesity and increases adiponectin expression	[161]
Wound healing effects	In vivo	Rat model	10 and 20% myricetin	○ Myricetin higher dose was effective in improving wound curing	[164]
Anti-allodynic effect	In vivo	Rat model	0.1–10 mg/kg	○ Myricetin decreased mechanical allodynia as well as thermal hyperalgesia	[168]
Anti-osteoporosis effects	In vivo	Rat model	1 or 2.5 mg/kg	○ Myricetin inhibited reduction in Bone mineral density and upregulated osteocalcin	[176]
	In vitro	MC3T3-E1 cells	20 μM	○ Myricetin promoted osteoblast differentiation and mineralization	[176]
	In vivo	Rat model	50 mg/kg	○ Myricetin treatment improves trabecular bone microarchitecture	[177]
Role in periodontitis and osteoporosis	In vivo	Mice model	2 or 5 mg/kg	○ Myricetin stopped alveolar bone resorption as well as increased alveolar crest height in the mouse model	[180]
Intraocular pressure-lowering activity	In vivo	Rabbit model	1 mg	○ Myricetin lowered IOP below control levels	[183]
Role in glaucoma	In vivo	Rat model	25, 50 or 100 mg/kg	○ Myricetin inhibited IOP elevation ○ It reduced inflammatory cytokines in the aqueous humor	[185]
Anti-osteoarthritis effects	In vitro	Mouse chondrocyte	12.5, 25 and 50 μM	○ Myricetin reduced inflammation and enhanced collagen-II generation	[190]
	In vivo	Mouse model	10 mg/kg	○ Myricetin mitigates the progression osteoarthritis	[190]
Renoprotective effects	In vivo	Mice model	3 mg/kg	○ Myricetin showed protective effects against nephrotoxicity	[66]
	In vivo	Mice model	50 and 200 mg/kg	○ Myricetin exerts nephroprotective activity by enhancing the renal antioxidative defense mechanisms	[193]
Role in the reproductive system	In vitro	VK2 and End1	5, 10, 20, 50 and 100 μM	○ Myricetin hampers cell growth and induces apoptosis ○ Myricetin inhibited cell proliferation	[194]

### 4.18. Role in the Reproductive System

The antigrowth potential of myricetin in endometriosis was tested. Myricetin inhibited cell cycle progression and cell proliferation of human VK2/E6E7 and End1/E6E7 cells and induction of apoptosis, with accumulation of reactive oxygen species, calcium ions, and the loss of mitochondrial membrane potential. Additionally, myricetin reduced lesion size in the endometriosis mouse model by inhibiting Ccne1. Therefore, myricetin exhibits antiproliferative effects on endometriosis through cell cycle regulation [194]. The role of myricetin in the reproductive system through modulation of different mechanisms is presented in Figure 7. Moreover, the wound-healing role of myricetin is summarized in Table 6. Relative uterus weights of rats in 100 mg/kg/day dose group of myricetin were increased according to vehicle control as well as positive control groups. Moreover, uterine heights were increased in positive control groups and myricetin 100 mg/kg/day dose group [195]. It was reported that myricetin treatment improved metabolic capacity as well as insulin sensitivity by activating BAT in dehydroepiandrosterone (DHEA)-induced polycystic ovary syndrome (PCOS) mice. Additionally, decreased cystic formation and an increased number of corpus luteum were observed in PCOS mice. Myricetin decidedly improved reproductive deficiencies in PCOS mice [196].

### 4.19. Anti-Microbial Effects

**Figure 8:**
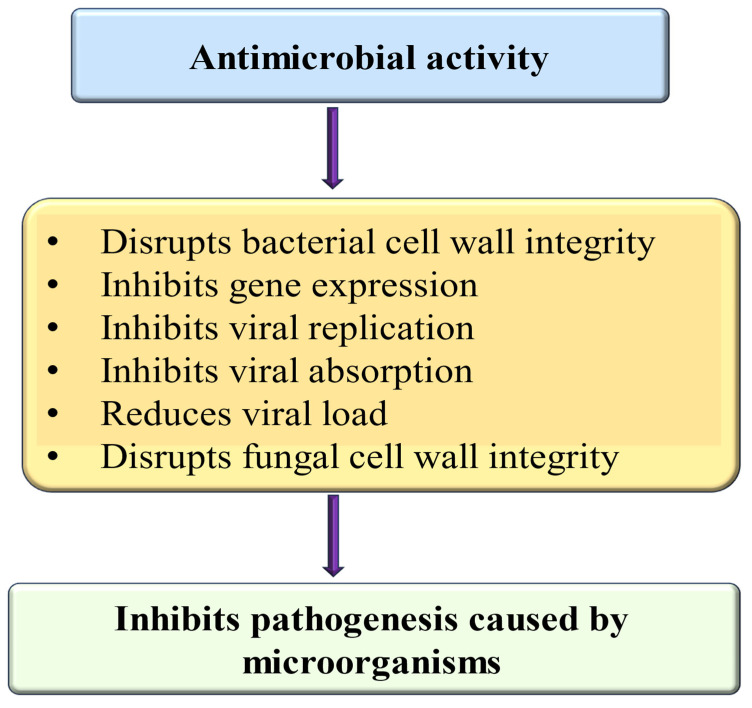
Antimicrobial potential of myricetin through different mechanisms.

Despite various initiatives aimed at decreasing consumption and regulating prescriptions, the prevalence of antibiotic resistance in clinical settings remains significant, and the emergence of bacterial pathogens with multidrug resistance is on the increase [197]. As a result, developing new approaches to combat antimicrobial resistance is essential for society. Focusing on the discovery of bioactive or natural compounds with antimicrobial properties could provide a sustainable solution to address this issue. The role of myricetin as an anti-bacterial, anti-fungal and anti-viral is presented in Figure 8. The role of myricetin microorganism-associated pathogenesis is summarized in Table 7. Antimicrobial potential, including antibacterial, antiviral, and antifungal properties, is explained here.

**I.**  Anti-bacterial potential

Myricetin oxidation products were generated via enzymatic oxidation of myricetin using horseradish peroxidase. It was reported that enzymatic oxidation enhanced the antibacterial activity and water solubility against *Staphylococcus aureus* of myricetin. Both myricetin and MYRoo could disturb the cell membrane, affect protein synthesis and degradation, and change the ROS levels in *S. aureus*. This study showed that enzymatic oxidation effectively improves MYR’s water solubility and antibacterial activity, enhancing its potential for food preservation [198]. Myricetin has been shown to inhibit the DnaB helicase of *E. coli* with a half-maximal inhibitory concentration (IC50) value of 11.3 μM. This crucial enzyme is vital for DNA replication and elongation [199]. A recent study was performed to assess the inhibitory potential of myricetin against Type II NADH oxidoreductase (NDH-2) as well as its effect on the expression and growth of virulence factors in *S. aureus*. It was demonstrated that myricetin was found to be an inhibitor of NDH-2 with a half-maximal inhibitory concentration of 2 μM. Time–kill assays exhibited that myricetin was a bactericidal agent against *S. aureus*. In line with being a competitive of NDH-2 substrate menadione (MK) inhibitor, the anti-staphylococcal potential of myricetin was antagonized through MK-4. Additionally, myricetin was shown to suppress the gene expression of enterotoxin SeA and decrease the hemolytic activity caused by *S. aureus* on rabbit erythrocytes in a dose-dependent way [200].

The effects of flavonols on recombinant sortase A (SrtA) as well as B (SrtB) prepared from *S. aureus* ATCC6538p were studied and it was noticed that these compounds inhibited the activity of sortases, without showing antibacterial activities. Among the flavonols tested, myricetin, morin, and quercetin showed strong sortase inhibitory activities [201]. Myricetin can effectively inhibit the activity of ClpP without affecting bacterial growth. Notably, myricetin reduced the pathogenicity of *Staphylococcus aureus* in vivo and enhanced the effectiveness of the traditional antibiotic oxacillin against methicillin-resistant Staphylococcus aureus (MRSA) infections. Additionally, it provided protection to mice from fatal lung infections caused by MRSA [202]. Myricetin notably decreases the production of various virulence factors in Staphylococcus aureus. This reduction encompasses diminished biofilm formation, adhesion, hemolysis, and staphyloxanthin production. Furthermore, myricetin offers significant protection against staphylococcal infections in the Galleria mellonella model [203]. Myricetin has been identified as an effective inhibitor of NDH-2, with a IC50 of 2 μM. The minimum inhibitory concentrations (MICs) of myricetin against *S. aureus* strains were found to be between 64 and 128 μg/mL. Furthermore, time-kill assays demonstrated that myricetin functions as a bactericidal agent against *S. aureus* [200] and myricetin demonstrated significant antimicrobial activity against foodborne pathogens [204].

**II.**  Anti-viral effects

The study was performed to assess the pharmacological efficiency and the mechanisms of action of myricetin against severe acute respiratory syndrome coronavirus 2 (SARS-CoV-2) infection both in vivo and in vitro. Myricetin meaningfully inhibited SASR-CoV-2 infection as well as replication in Vero E6 cells. Furthermore, myricetin showed a noticeable suppressive action on the receptor-interacting serine/threonine protein kinase 1 (RIPK1)-driven inflammation as well as NF-kappa B signaling in THP1 macrophages. In animal model studies, myricetin remarkably ameliorated carrageenan-caused paw edema in rats, delayed-type hypersensitivity (DTH) induced auricle edema in mice, and LPS-induced acute lung injury (ALI) in mice [205]. Myricetin demonstrated strong antiviral effects against the pseudorabies virus (PRV) by directly inactivating the virus in vitro and hindering its adsorption, replication, and penetration into cells. In a mouse model infected with PRV, myricetin treatment enhanced the survival rate by 40% five days after infection. Furthermore, treatment with myricetin reduced the pathological changes associated with PRV infection [206]. Papain-like protease (PLpro) was selected as the target to screen antiviral agents against infectious bronchitis virus (IBV). It was reported that myricetin exhibited the robust inhibitory effect on IBV PLpro. Moreover, myricetin suggestively inhibits IBV replication in primary chicken embryo kidney cells and it can upregulate the transcription levels in the IRF7 and NF-κB signaling pathways [207]. It was reported that myricetin effectively inhibited transmissible gastroenteritis virus (TGEV)-induced cytopathic effects in a dose-dependent way. Myricetin meaningfully reduced TGEV viral load and at 100 μM concentration directly inactivated TGEV and suppressed its intracellular replication stage. In addition, treating PK-15 cells with 100 μM myricetin demonstrated a protective benefit against TGEV infection. Myricetin acted as a competitive inhibitor of PLpro, exhibiting an IC50 value of 6.563 μM [208]. The findings revealed that myricetin demonstrated anti-HSV-1 and HSV-2 properties with minimal toxicity, outperforming the effects of acyclovir [209].

**III.**  Anti-fungal effects

Heung-Shick Lee and Younhee Kim, 2022 reported that myricetin showed antifungal activity against *Candida albicans* via damaging the cell wall integrity and remarkably increasing the membrane permeability [210]. In vitro and vivo, myricetin’s anti-biofilm effect and its ability to increase miconazole nitrate’s (MN) antifungal effects were investigated. Myricetin exhibited a significantly inhibitory effect on biofilm formation and myricetin showed no effect on *Candida albicans* cell viability. MIC_50_ and MIC_80_ of MN were correspondingly reduced when used in combination with myricetin. Myricetin efficiently prevented the mouse periprosthetic joint infection (PJI), and MN was incorporated into thermosensitive hydrogel (TSH) [211].

**Table 7:** Anti-microbial potential of myricetin.

Activities	Species	Key Findings	Ref.
Anti-bacterial	*Staphylococcus aureus*	○ Myricetin disrupts cell membrane integrity, affects protein synthesis as well as degradation	[198]
	*Escherichia coli*	○ Myricetin inhibits DnaB helicase	[199]
	*S. aureus*	○ Myricetin causes sortase inhibitory activities	[201]
	*S. aureus*	○ Myricetin showed bactericidal agent	[200]
	*SARS-CoV-2*	○ Myricetin inhibited SARS-CoV-2 replication	[205]
Anti-viral	*Pseudorabies virus*	○ Myricetin directly inactivates the virus, inhibiting viral adsorption, cell penetration and replication,	[206]
	*Transmissible gastroenteritis virus*	○ Myricetin inhibited TGEV-induced cytopathic effects. ○ Myricetin reduced TGEV viral load and inactivated TGEV	[208]
	*Herpes simplex virus*	○ Myricetin blocks HSV infection and ○ down-regulates the cellular EGFR/PI3K/Akt signaling pathway	[209]
	*Candida albicans*	○ Myricetin disturbs the cell wall integrity and membrane permeability increased	[210]
Anti-fungal	*Candida albicans*	○ MIC_50_ and MIC_80_ of miconazole nitrate decreased when combined with myricetin	[211]

## 5. Synergetic Effects of Myricetin with Other Drugs

Natural compounds and their bioactive components contribute to disease management mainly by exhibiting antioxidant and anti-inflammatory properties, as well as by influencing various biological functions. The impact of foods on human biological systems varies significantly when consumed as part of a whole matrix, compared to the effects of individual food constituents [212]. Physiological synergy occurs when plant compounds work together to enhance absorption, bioavailability, and metabolism while also reducing potential adverse effects [213]. Studies have indicated that drug combinations can improve effectiveness through different mechanisms, such as boosting antioxidant, anti-inflammatory, and anti-cancer properties while minimizing the side effects or toxicity associated with the individual drugs. Synergetic effects of myricetin with other drugs are summarized in Table 8. CuMy-12 (Cucurbitacin E (CuE) and myricetin (Myr), a combination of CuE as well as Myr from C. colocynthis, inhibited cell proliferation and caused cell cycle arrest in the G0/G1 phase and induced apoptosis, revealing a synergistic effect [214]. The effect of the combination of myricetin with vancomycin and oxacillin against vancomycin intermediate resistant *S. aureus* (VISA) and methicillin resistant *S. aureus* (MRSA) strains was investigated. Checkerboard assessments showed a decrease in the minimum inhibitory concentration (MIC) of antibacterials when myricetin was present, while time-kill tests confirmed the synergistic effects of these combinations, with the exception of the VISA strain when myricetin was combined with vancomycin. In vivo assessments demonstrated that the combination of oxacillin and myricetin was effective in treating MRSA-infected larvae, leading to a 20% increase in host survival compared to the control groups [215]. As per the results of the microdilution test, the bacterial strains used in this study (except for MRSA) were commonly sensitive to antibiotics. The interaction study revealed encouraging findings about the synergistic effects of antibiotics combined with flavonoids. Notably, myricetin demonstrated a synergistic interaction exclusively with levofloxacin [216]. A study was made to investigate the combined anti-diabetic effect of myricetin and kaempferol in streptozotocin (STZ)-activated diabetes in rats. The study demonstrated that combined treatment restored the measured parameters including inflammatory cytokines, glucose levels, oxidative markers, and liver and lipid enzymes in diabetic rats. The findings indicate that the combined treatment of myricetin and kaempferol offers positive effects against diabetes, suggesting that this combination could be therapeutically effective in managing the condition [217]. To investigate whether myricetin (MYR) and methyl eugenol (MEG) can enhance the anticancer effects of cisplatin (CP) on cervical cancer cells, HeLa cells were treated with MYR and MEG either individually or in combination with cisplatin and then assessed for cell growth and apoptosis. The results indicated that the combination of MYR or MEG with CP was more effective in suppressing cancer cell growth and promoting apoptosis compared to treatment with each drug alone. Furthermore, the loss of mitochondrial membrane potential (ΔΨm) and Caspase-3 activity were substantially greater in the combination treatment than in the single drug treatment [218]. Another study was executed to examine whether sulforaphane (SFN) and myricetin (Myr) synergistically induce apoptosis in adipocytes. As compared with the effects of each compound alone, the combination of SFN and Myr synergistically induced apoptosis, and reduced cell viability [219]. It was established that myricetin showed a strong affinity for CD147 as well as down-regulating the protein level of CD147 by facilitating its proteasome-dependent degradation. Furthermore, synergistic antitumor effects of myricetin and cisplatin were noticed in both in vivo and in vitro [220].

## 6. Myricetin-Based Nanoformulation and Its Role in Disease Management

**Figure 9:**
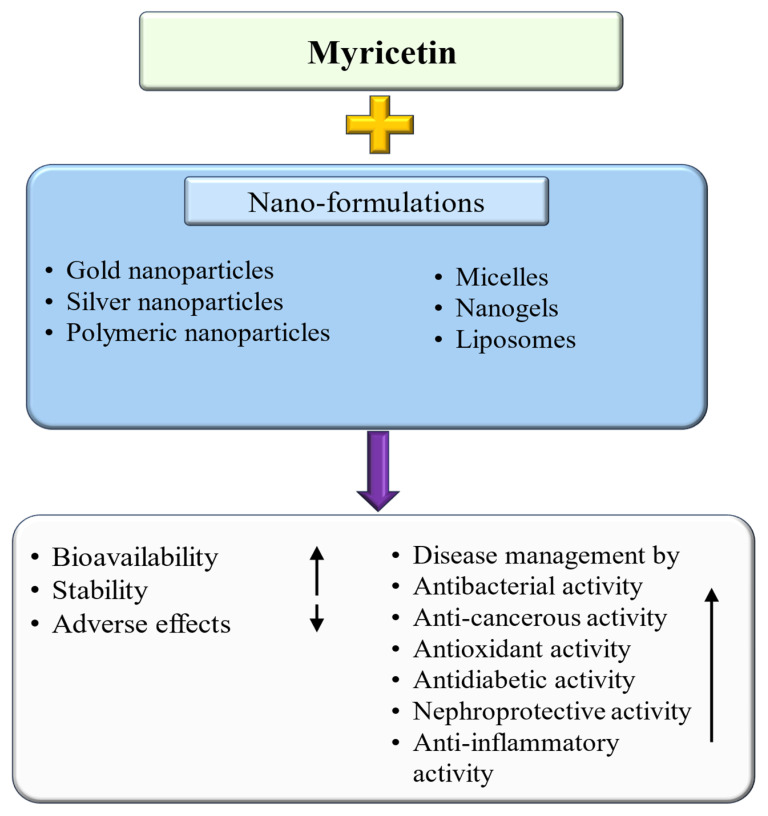
Myricetin-based nanoformulation and their role in disease management. The downward-pointing arrow shows downregulation, whereas the upward arrow represents upregulation.

Although myricetin is beneficial in managing pathogenesis, its main challenge is low bioavailability. However, various nanoformulations have been developed to enhance the bioavailability, depletion of toxicity, solubility, and targeted delivery of drugs. Nanoformulations also enhance the pharmacokinetic properties of bioactive compounds, leading to improved therapeutic effects. Moreover, nanoformulations can be introduced into the body through inhalation, ingestion, skin penetration, or injection, interacting with biological systems [222]. Additionally, nanoformulations can reduce the required administration doses by enabling targeted delivery and site-specific release, thereby enhancing the therapeutic effects of drugs [223]. The role of myricetin-based nanoformulations in disease are presented in Figure 9 and Table 9. A study was performed to synthesize the silver nanoparticles labelled with myricetin. It was reported that myricetin was capable of killing human colorectal cancer cell lines [224]. Myricetin-mediated silver nanoparticles (MY-AgNPs) were produced. These nanoparticles showed potent antibacterial activity against *Salmonella* and *Escherichia coli*, with minimum inhibitory concentrations of 10^−4^ and 10^−5^ g/L, respectively. Moreover, MY-AgNPs showed better antioxidant potential [225]. The gold nanoparticles (AuNPs) with myricetin (Myr) were synthesized and Myr-AuNPs-treated cells presented a good proportion of dead cells demonstrated with formation of pro-apoptotic bodies [226]. The study presents liposomal nanoformulations of myricetin showed to enhance its bioavailability while minimizing pro-oxidant activity. The impact of nanoencapsulated myricetin on developing zebrafish embryos was investigated. Results from the cumulative hatchability, developmental studies, and antioxidant assays indicated that the liposomal nanoformulation of myricetin exhibited enhanced antioxidant activity, offering protection against oxidative stress [227]. The study was planned to promote bioavailability as well as the therapeutic efficacy of myricetin through pro-liposome formulations. The better pharmacological effects, particularly the hepatoprotective and antioxidant activity of myricetin, were achieved through TPGS-modified pro-liposome formulation [228]. Myricetin was incorporated into pH-sensitive liposomes to improve its bioavailability and anti-hyperuricemic activity. The release rate of myricetin liposomes exceeded that of free myricetin, with the cumulative value responding to pH. Additionally, the maximum concentration (Cmax) of myricetin liposomes was measured at 4.92 ± 0.20 μg/mL. In the M-L-H group (200 mg/kg), the uric acid level decreased by 54.74% *w*/*v* compared to the model group [229]. High-bioavailability liposomes encapsulating dihydromyricetin (DHM) were developed and evaluated for their therapeutic effects and regulatory mechanisms in a model of hepatic inflammatory injury. The encapsulation of DHM in liposomes enhanced its bioavailability, resulting in a more effective reduction in liver inflammation. Additionally, DHM liposomes specifically targeted hepatic macrophages and polarization of macrophages into an anti-inflammatory phenotype [230]. A study was performed to formulate myricetin in the form of solid lipid nanoparticles (SLN), decorated with chitosan (CS) as well as active-targeted with folic acid (FA). The cytotoxicity assay displayed that myricetin-solid lipid nanoparticles (SLN)-chitosan (CS)- folic acid (FA) killed cancer cells, and the highest level of apoptosis was shown at the concentration of 45 µg/mL. The anti-angiogenic activities of the formulations showed a decrease in the number and length of the vessels and also affected VEGFR and VEGF genes involved in angiogenesis. The antitumor studies designated the effects of formulation on reducing tumor volume [231]. The myricetin nanofiber (MyNF) system, which combines hydroxypropyl-β-cyclodextrin (HPBCD) and polyvinylpyrrolidone K120 (PVP), has been developed to load myricetin, enhancing its water solubility and skin penetration by altering its physicochemical properties. The results revealed that these myricetin nanofibers significantly reduced cytotoxicity in HaCaT keratinocytes when compared to free myricetin [232].

The nanoemulsion of Myricetin (Myr-NE) was developed and assess its benefit over myricetin alone in Triple-negative breast cancer (TNBC) cells. It was reported that Myr-NE showed noteworthy inhibition of cell proliferation, clonogenicity, and increased apoptosis with ~2.5-fold lower IC50 as compared to myricetin. Mechanistic study demonstrated that nanoemulsion improved the anti-cancer effectiveness of Myricetin, probable through inhibiting the PI3K/AKT/mTOR pathway, ultimately leading to enhanced cell death in TNBC cells [233]. A nanoformulation was prepared with myricetin encapsulated within chitosan nanoparticles (CHT-NPs) such as MYR-CHT-NPs. In vivo, pharmacodynamic study followed by oral administration in rats exhibited better glycaemic control than existing drugs. The nanoparticles also showed a controlled increase in weight compared to Metformin. The nanoformulation reduced the levels of several pathological biomarkers and showed no toxicity or changes in the section of the organ as compared to normal control, suggesting safe oral administration of the encapsulated myricetin [234]. A Kolliphor HS15-based myricetin-loaded (HS15-Myr) nanomicelle was prepared to evaluate its effectiveness against cisplatin-induced acute kidney injury (AKI). In vitro results indicated that this formulation improved the antioxidant activity of myricetin and countered the proliferation inhibition of HK-2 cells caused by cisplatin. Additionally, the HS15-Myr nanomicelles effectively reduced the accumulation of reactive oxygen species, DNA damage, and decreased mitochondrial membrane potential induced by cisplatin. In an in vivo study involving mice, the results showed that the formulation reversed the adverse effects of cisplatin, including reductions in renal indices and body weight, as well as increased levels of blood urea nitrogen and serum creatinine. Moreover, the pretreatment with the nanomicelles significantly affected the activities of antioxidant enzymes altered by cisplatin. Furthermore, the formulation successfully inhibited the inflammatory responses triggered by cisplatin in the kidney tissue of mice [235]. A study developed a myricetin-coated zinc oxide nanocomposite, reporting that the tested C. perfringens isolates were sensitive to myricetin-loaded zinc oxide (ZnO)/polyvinyl alcohol (PVA), with minimum inhibitory concentrations ranging from 0.125 to 2 µg/mL [236].

**Table 9:** Myricetin-based nanoformulation and their role in disease.

Nanoformulations	Activity	Key Findings	Ref.
Myricetin-mediated silver nanoparticles	Antibacterial	○ Nanoformulation showed good antibacterial activity against *E. coli* and *Salmonella*	[225]
Myricetin-gold nanoparticles	Anti-cancerous	○ Nanoformulation-treated cells exhibited a good proportion of dead cells, depolarization of mitochondrial membrane potential	[226]
Myricetin liposomal nanoformulation	Antioxidant	○ Liposomal nanoformulation of myricetin showed enhanced antioxidant activity	[227]
TPGS modified pro-liposome of myricetin	Hepatoprotective	○ A good antioxidant and hepatoprotective activity, achieved rather than the TPGS-free pro-liposome as well as unformulated MRC	[228]
MYR pH-sensitive liposomes	Anti-hyperuricemic	○ Myricetin liposomes showed pH sensitivity and enhanced the oral bioavailability and anti-hyperuricemic efficacy of myricetin	[229]
Myricetin-loaded solid lipid nanoparticles	Anti-cancerous	○ This formulation showed higher toxicity on cancer cells than on free myricetin	[231]
Myricetin nanofibers	Photoprotective	○ Myricetin nanofibers can decrease cytotoxicity in keratinocytes compared to free myricetin	[232]
Myricetin encapsulated chitosan nanoformulation	Diabetes management	○ The formulation showed an attractive delivery vehicle in improving the blood glucose level with controlled weight	[234]
Myricetin-loaded nanomicelles	Nephroprotective	○ These nano micelles enhanced the antioxidant activity of myricetin	[235]

## 7. Conclusions and Future Perspectives

Around 80% of the global population depends on plant-based extracts for their primary healthcare needs, highlighting the vital role of traditional medicine in various cultures. These natural remedies are often the first line of defense against various pathogenesis, providing relief and health-promoting potential. Natural products, including stems, fruits, leaves, and seeds, play a significant role in disease management by modulating various biological activities. Flavonoids have also been demonstrated to significantly contribute to disease prevention due to their antioxidant and anti-inflammatory properties.

Flavonoids have emerged as important agents in disease prevention, largely due to their powerful antioxidant and anti-inflammatory properties. These compounds help combat oxidative stress by neutralizing free radicals, which can lead to cellular damage and numerous chronic diseases. Their anti-inflammatory effects also contribute to the decrease of inflammation-related pathogenesis. By modulating various biochemical and physiological pathways, flavonoids increase the body’s defense mechanisms. Based on evidence from in vitro and in vitro studies, flavonoids play a significant role in disease cure through various mechanisms. Myricetin is a member of the class of flavonoids/plant-derived flavonoids known as flavonols and is commonly found in vegetables and fruits. The amount present in various fruits and vegetables varies within different ranges. Its role in disease management is confirmed through potential mechanisms, including anti-inflammatory and antioxidant effects, lowering lipid and cholesterol levels, enhancement of insulin, and preservation of the structural integrity of tissues. This review emphasizes the recent findings regarding myricetin and its potential use in different diseases. Further research with adequate, randomized, and controlled clinical trials should be performed to explore this compound’s therapeutic importance, safety, and efficacy against human pathogenesis.

## Figures and Tables

**Table 1:** Hepatoprotective activities of myricetin through different mechanisms.

Activity	Study Types	Dose	Outcome	Ref.
Hepatoprotective effect	In vivo, mice model	50 mg/kg	○ Myricetin treatment reduced liver fibrosis ○ Decreased the increase in liver function enzyme activity	[78]
	In vivo, mice model	100 mg/kg	○ Myricetin improved hepatic steatosis and increased hepatic type 1 deiodinase activities.	[79]
	In vivo, mice model	25, 50, 100 mg/kg	○ Myricetin protected fulminant hepatitis by improving oxidative stress and lowering AST and ALT levels ○ Histopathological changes, hepatic apoptosis, and inflammation improved	[80]
	In vivo, mice model	25 or 100 mg/kg	○ Myricetin decreased the fatality rate as well as pathological liver changes and improved liver function enzymes ○ Decreased apoptotic, inflammatory factors and oxidative	[81]
	In vivo, rat model	25, 50 mg/kg	○ Myricetin inhibited hepatotoxicity and reduced the production of free radicals and inflammatory markers ○ Lipid membrane integrity and liver tissue architecture was maintained by myricetin	[19]
	In vivo, rat model	HFD containing 0.5% myricetin	○ Myricetin reduced hepatic lipid synthesis and inflammation	[82]
	In vivo, mice model	100, 200 mg/kg	○ Myricetin pretreatments increased liver antioxidant enzyme activities and decreased liver function enzymes and inflammatory markers ○ Myricetin administration maintained the liver architecture	[83]
	In vivo, mice model	25, 50, 100 mg/kg	○ Myricetin pretreatments increased antioxidant enzyme activities and decreased inflammatory markers	[44]
	In vivo, mice model	Diet containing 0.04% or 0.08% myricetin	○ Regulation of hepatic lipid metabolism transcription factors and pro-inflammatory cytokines by myricetin	[84]
	In vivo, mice model	100 mg/kg	○ Myricetin-treated prevented liver fibrosis	[17]
	In vitro, LPS-stimulated RAW264.7 macrophages	50 μM	○ Myricetin inhibited mRNA expression of M1 macrophage marker genes	[17]
	In vivo, rats	25, 50 mg/kg	○ Myricetin prevented hepatotoxicity by modifying the inflammatory markers and production of free radicals ○ Myricetin maintained oxidant-antioxidant status and lipid membrane integrity	[19]

**Table 5:** The potential role of myricetin in prevention of cancer.

Cancer	Modulation	Study Types	Model	Mechanism	Outcome	Refs.
Ovarian	Angiogenesis	In vitro	OVCAR-3 and A2780/CP70	VEGF ↓ Angiogenesis ↓	○ Myricetin acted as an anti-angiogenic agent ○ Myricetin reduced VEGF secreted by cancer cells ○ Myricetin inhibited in vitro angiogenesis induced by cancer cells	[131]
Breast	Angiogenesis	In vitro and in vivo	MDA-MB-231,4T1 & in vivo tumor xenograft model	VEGF ↓ VEGFR ↓ Angiogenesis ↓	○ Myricetin reduced vascular endothelial growth factor levels ○ Myricetin downregulated p38MAPK and VEGFR2.	[132]
Breast	Nrf-2/GPX4 pathway	In vitro and in vivo	Breast tumors mice model 4 T1	Nrf-2 ↓, GPX4 ↓	○ Myricetin induced ferroptotic 4 T1 cell death through downregulating GPX4 and Nrf-2 ○ Myricetin reduced the growth of subcutaneous breast tumors evidenced by inhibiting Nrf-2 and GPX4 expression	[133]
Gastric	Apoptosis	In vitro and in vivo	AGS & Xenograft	Bcl-2/Bax ratio ↓ Bax ↑ Bcl2 ↓	○ Myricetin induced apoptosis	[134]
Gastric	PI3K/Akt/mtor	In vitro	AGS	p-PI3K, p-Akt and p-mTOR ↓	○ Myricetin induced apoptosis by PI3K/Akt/mTOR pathway inhibition	[134]
Gastric	Apoptosis	In vitro	In vitro	Bcl-2 ↓ and pro-caspase-3 Bax and cleaved caspase-3 ↑	○ Myricetin induced apoptosis and influenced apoptosis by regulating related proteins	[135]
Pancreatic cancer	Apoptosis	In vitro	MIA PaCa-2, Panc-1 and S2-013	Caspase-3 and 9 ↑ Induction of apoptosis	○ Myricetin treatment increased apoptosis in pancreatic cancer cells	[136]
Brain	Apoptosis	In vitro	U251	Bax and Bad levels ↑ Bcl-2 and Bcl-xl ↓	○ Myricetin induced cell apoptosis in human glioma cells	[137]
Liver	Cell cycle	In vitro	Hep3B and HepG2i	Blocking cell cycle at the G2/M phase cell number in the G0/G1 phase ↓	○ Myricetin arrested cell cycle ○ Reduced liver cancer proliferation	[138]
Liver	Cell cycle	In vitro	HepG2	Accumulation of cells in the G2/M phase ↑ Protein levels of the p53/p21 cascade ↑	○ Myricetin caused HepG2 cell arrest at the G2/M phase	[139]
Liver	Autophagy	In vitro	SMMC-7721 and Hep3B	Ratio of LC3-II/LC3-I ↑ autophagic flux ↑	○ Myricetin induced autophagy	[140]
Ovarian	PI3K/AKT	In vitro	A2780 and HO8910	Phosphorylated ERK and PI3K/AKT ↓	○ Myricetin suppressed the phosphorylation levels in the PI3K/AKT and MAPK/ERK signaling pathways.	[141]
Bile duct	STAT3 pathway	In vitro	KKU-100	STAT3 ↓	○ Treatment of cells with myricetin-suppressed STAT3 phosphorylation	[142]

The downward-pointing arrow shows downregulation, whereas the upward arrow represents upregulation.

**Table 8:** Synergistic effects of myricetin with other drugs.

	Drugs/Compound	Activity	Key Finding	Ref.
Myricetin	Cucurbitacin E	Anti-lung cancer	○ The combination of cucurbitacin E and myricetin inhibited cell proliferation and colony formation, induced apoptosis and cell cycle arrest	[214]
	Oxacillin	Antibacterial	○ When myricetin was combined with oxacillin, the MRSA strain became susceptible to the antibacterial	[215]
	Levofloxacin	Antibacterial	○ Myricetin showed synergistic interaction only with levofloxacin	[216]
	Kaempferol	Anti-diabetic	○ The combined treatment restored inflammatory cytokines, glucose levels, lipid and liver enzymes and oxidative markers	[217]
	Methyl eugenol	Anti-cancerous	○ The combined treatment led to an increased number of cells in the G0/G1 phase	[218]
	Sulforaphane	Anti-obesity	○ FN plus Myr was a more potent inducer of apoptosis than either compound alone	[219]
	Cisplatin	Anti-cancerous	○ Synergistic antitumor effects of myricetin and cisplatin was noticed	[220]
	Piceatannol	Anti-cancerous	○ Piceatannol and myricetin synergistically induced apoptosis in cancer cells	[221]

## List of Abbreviations

GSH	Glutathione
T-AOC	Total antoxidant capacity
SOD	Superoxide dismutase
CAT	Catalase
MDA	Malondialdehyde
NAFLD	Nonalcoholic fatty liver disease
ROS	Reactive oxygen species
AGEs	Advanced glycation end-products
Nrf2	Nuclear factor erythroid 2–related factor 2
iNOS	Inducible nitric oxide synthase
ALP	Alkaline phosphatase
ALT	Alanine transaminase
AST	Aspartate aminotransferase
COX-2	Cyclooxygenase-2
TNF-α	Tumor necrosis factor-α
NF-κB	Nuclear factor kappa-light-chain-enhancer of activated B cells
IL	Interleukin
STAT	Signal transducer and activator of transcription
MMP	Matrix metalloproteinase
Cmax	Maximum concentration
IBD	Inflammatory bowel diseases
5-FU	5-Fluorouracil
CCl4	Carbon tetrachloride
LPS	Lipopolysaccharide
BALF	Bronchoalveolar Lavage Fluid
STZ	Streptozotocin
HFD	High-fat diet
MAPK	Mitogen-activated protein kinase
TLR	Toll-like receptor
OA	Osteoarthritis
BUN	Blood Urea Nitrogen
VEGF	Vascular endothelil growth factor
PTZ	Pentylenetetrazole
PCO	Polycystic Ovary Syndrome
HIF-1α	Hypoxia-inducible factor-1α
IOP	Intraocular pressure
MPO	Myeloperoxidase
POAG	Primary open-angle glaucoma
MIC	Minimum inhibitory concentration
AuNP	Gold nanoparticle
AgNP	Silver nanoparticle
SLN	Solid nanoparticle
CDAHFD	Choline-deficient, L-amino acid-defined, high-fat diet
HSC	Hepatic stellate cells
NASH	Nonalcoholic steatohepatitis
TREM-1	Triggering receptor expressed on myeloid cells-1
Pi3k/Akt/mTOR	Phosphoinositide 3 kinase/Akt/mTOR
CDK	Cyclin-dependent kinase
Fe-NTA	Ferric iron nitrilotriacetate
GSH-Px	Glutathione peroxidase
TBARS	Thiobarbituric acid reactive substance
PGN	Peptidoglycan
mTOR	Mammalian target of rapamycin
